# Santalol Isomers Inhibit Transthyretin Amyloidogenesis and Associated Pathologies in *Caenorhabditis elegans*

**DOI:** 10.3389/fphar.2022.924862

**Published:** 2022-06-16

**Authors:** Amirthalingam Mohankumar, Duraisamy Kalaiselvi, Govindhan Thiruppathi, Sivaramakrishnan Muthusaravanan, Subramaniam Vijayakumar, Rahul Suresh, Shinkichi Tawata, Palanisamy Sundararaj

**Affiliations:** ^1^ PAK Research Center, University of the Ryukyus, Okinawa, Japan; ^2^ Department of Zoology, Bharathiar University, Coimbatore, India; ^3^ Department of Agricultural Chemistry, Institute of Environmentally Friendly Agriculture, College of Agriculture and Life Science, Chonnam National University, Gwangju, South Korea; ^4^ Department of Biotechnology, Mepco Schlenk Engineering College, Sivakasi, India; ^5^ Department of Medical Physics, Bharathiar University, Coimbatore, India; ^6^ International Research Center of Spectroscopy and Quantum Chemistry—IRC SQC, Siberian Federal University, Krasnoyarsk, Russia

**Keywords:** transthyretin, familial amyloid polyneuropathy, santalol isomers, synergism, *Caenorhabditis elegans*, tetramer stabilizer

## Abstract

Transthyretin (TTR) is a homotetrameric protein found in human serum and is implicated in fatal inherited amyloidoses. Destabilization of native TTR confirmation resulting from mutation, environmental changes, and aging causes polymerization and amyloid fibril formation. Although several small molecules have been reported to stabilize the native state and inhibit TTR aggregation, prolonged use can cause serious side effects. Therefore, pharmacologically enhancing the degradation of TTR aggregates and kinetically stabilizing the native tetrameric structure with bioactive molecule(s) could be a viable therapeutic strategy to hinder the advancement of TTR amyloidoses. In this context, here we demonstrated α- and β-santalol, natural sesquiterpenes from sandalwood, as a potent TTR aggregation inhibitor and native state stabilizer using combined *in vitro*, *in silico*, and *in vivo* experiments. We found that α- and β-santalol synergize to reduce wild-type (WT) and Val30Met (V30M) mutant TTR aggregates in novel *C. elegans* strains expressing TTR fragments fused with a green fluorescent protein in body wall muscle cells. α- and β-Santalol extend the lifespan and healthspan of *C. elegans* strains carrying TTR_WT_::EGFP and TTR_V30M_::EGFP transgene by activating the SKN-1/Nrf2, autophagy, and proteasome. Moreover, α- and β-santalol directly interacted with TTR and reduced the flexibility of the thyroxine-binding cavity and homotetramer interface, which in turn increases stability and prevents the dissociation of the TTR tetramer. These data indicate that α- and β-santalol are the strong natural therapeutic intervention against TTR-associated amyloid diseases.

## Introduction

Hereditary amyloidogenic transthyretin (ATTR) amyloidosis with polyneuropathy, commonly known as transthyretin-type familial amyloid polyneuropathy (FAP), is a sparse condition mostly found in Japan, France, Portugal, Sweden, Spain, and descendants of these regions (Coelho et al., 2013). FAP is a life-limiting, autosomal dominant, severe systemic disorder with predominant somatic and autonomic peripheral nervous system involvement caused by a mutation in the transthyretin gene (*TTR*) (Benson et al., 2020). FAP is characterized by the extracellular deposition of amyloid fibril containing insoluble mutant TTR variants. Amyloid fibril deposition can be observed in autonomic and peripheral nerves, heart, intestine, lungs, and carpal tunnel of affected individuals. Familial amyloid cardiomyopathy (FAC) and senile systemic amyloidosis (SSA) are also caused by the misfolding and subsequent aggregation of mutant and wild-type TTR, respectively. Hereditary amyloidosis can be of the wild type (ATTRwt) or the mutated form (ATTRm). ATTRwt is the most common age-related type of amyloid disease, and ATTRm occurs due to various point mutations (Pinney et al., 2013). Over 140 different amyloidogenic TTR variants have been reported (Rowczenio et al., 2014). TTR is a 55 kDa homotetramer consisting of four identical 14 kDa monomers with 127 amino-acid residues each and is rich in β-sheets with four binding sites; two for retinol (vitamin A)-retinol-binding protein (RBP) complex and two for thyroxine (T_4_). TTR transports T_4_ and the retinol-RBP complex to the brain and various body parts (Hörnberg et al., 2000). Two funnel-shaped T_4_ binding pockets are located at the center of the tetramer, which contributes to the stability of the TTR by binding with T_4_ between two dimers. Tetramer dissociation is the rate-limiting step for TTR amyloid fibril formation. Accordingly, point mutations destabilize the native tetrameric TTR and thus increase amyloidogenesis (Hammarström et al., 2002). Therefore, stabilizing TTR tetramer through pharmacological interventions is a promising strategy for treating TTR amyloidoses.

For many years, heart or heart-liver combined transplantation and prophylactic pacemaker implantation have been the only palliative treatments for TTR amyloidosis (Holmgren et al., 1991). Recently, it was shown that several bioactive phytomolecules and small molecules could stall TTR amyloidogenesis. These molecules are known to bind strongly to the unoccupied T_4_-binding sites within TTR and kinetically stabilize the native quaternary structure, which limits the tetramer destabilization and aggregate formation (Adams et al., 2019; Yokoyama and Mizuguchi, 2020). Mounting clinical and preclinical evidence indicated that tafamidis and diflunisal reduced the progression of early- and late-onset ATTRwt and Val30Met (ATTR_V30M_) amyloidoses and diseases associated with other TTR variants (Bulawa et al., 2012; Berk et al., 2013). Sant’Anna and others found that tolcapone, an FDA-approved molecule for Parkinson’s disease, inhibits TTR aggregation and toxicity, binds to TTR in human plasma, and stabilizes the tetramer dissociation in mice and humans (Sant’Anna et al., 2016). Several natural compounds have been shown to prevent the early stage of TTR tetramer dissociation and inhibit specific TTR fibril formation (Ciccone et al., 2020; Yokoyama and Mizuguchi, 2020; Matsushita et al., 2021). These observations clearly indicated that the pharmacological enhancement of the breakdown of TTR aggregates and kinetic stabilization of the native tetrameric structure of TTR with bioactive molecules could be a viable therapeutic strategy to hinder the advancement of these diseases. Santalol isomers (α- and β-santalol), the active ingredients in *Santalum album* L. essential oil, seem promising in this endeavor. Our previous study found that santalol isomers offer stress-resilience, extend healthy longevity, and retard age-related functional deficits in *C. elegans* (Mohankumar et al., 2018; Mohankumar et al., 2019b). Importantly, santalol isomers reduced the protein aggregates strongly associated with pathologies of Alzheimer’s, Parkinson’s, and Huntington’s diseases by activating multiple cellular signaling pathways (Mohankumar et al., 2018; Mohankumar et al., 2020). Here, we identify santalol isomers as a potential inhibitor of TTR amyloidogenesis. We have shown that α- and β-santalol act synergistically to reduce the aggregation and toxicity of wild-type and V30M mutant TTR and to alleviate other TTR-associated pathologies by selectively activating SKN-1/Nrf2, autophagy, and proteasome more effectively than the orally active TTR inhibitor tolcapone. Moreover, α- and β-santalol binds to the T_4_-binding site and stabilizes the wild-type and V30M mutant TTR, thereby preventing the tetramer dissociation.

## Materials and Methods

### Materials

α- and β-Santalol were purified from sandalwood (*Santalum album* L.) oil as described (Mohankumar et al., 2018). Nematode and bacterial growth media and their components, antibiotics, sodium hydroxide (NaOH), household bleach (NaClO), sodium azide (NaN_3_), and dimethyl sulfoxide (DMSO) were purchased from HiMedia Laboratories Pvt. Ltd. (Mumbai, India). Tolcapone, chloroquine, Epigallocatechin gallate (EGCG), isopropyl *β*-D-1-thiogalactopyranoside (IPTG), 5-fluoro-2′-deoxyuridine (FUdR), 2′,7′-dichlorodihydrofluorescein diacetate (H_2_DCF-DA), prealbumin from human plasma, and MG-132 were obtained from Sigma Aldrich (St. Louis, MO, United States).

### *C. elegans* Strains, Genetics, and Handling

*C. elegans* were cultured on standard nematode growth media (NGM) agar plates carrying *E. coli* OP50 and maintained at 20°C using the standard techniques (Brenner, 1974). The wild-type (N2), *hlh-30*[*tm1978*], and *bec-1*[*ok700*] strains were obtained from the *Caenorhabditis* Genetics Centre (CGC, University of Minnesota, MN, United States). The novel strains expressing several type of TTR fragments fused with GFP in body wall muscle cells *viz*., zyeEx1069[pCKX3307(*unc-54p::nsTTR*_*WT*_ *::EGFP*)], zyeEx1070[pCKX3311(*unc-54p::nsTTR*_*1-80*_ *::EGFP*)], zyeEx1073[pCKX3303(*unc-54p::nsTTR*_*81-127*_ *::EGFP*)], zyeEX1076[pCKX3316(*unc-54p::nsEGFP*)], zyeEx1078[pCKX3309(*unc-54p::nsTTR*_*V30M*_ *::EGFP*)], and zyeEx1082[pCKX3313(*unc-54p::nsTTR*_*49-127*_ *::EGFP*)] were gifted by Professor Kunitoshi Yamanaka (Kumamoto University, Japan) (Tsuda et al., 2018). Synchronized culture of worms were obtained by alkaline hypochlorite treatment of gravid adult worms as described previously (Stiernagle, 2006).

### Lifespan Analysis

Lifespan was measured at 20°C as previously described (Mohankumar et al., 2020). In brief, synchronized L1 stage worms (10–15 worms per plate for a total of 100–120 individuals per experiment) were raised on plates supplemented with santalol isomers and tolcapone. Worms were scored as live or dead, and surviving nematodes were transferred to fresh plates every day during the reproduction period and 4–5 days thereafter. Animals were censored from the analysis if they suffered from internal hatching, desiccated on the edge of the plate, ruptured, mechanical death, or lost. Death was confirmed and scored based on the failure to respond to mild contact with a metal worm picker and loss of pharynx movement.

### Phenotype Assay

To measure the body bends, control and santalol isomers treated worms were gently washed off from the plates and released on the agar plates to crawl for 5 min at 20°C. Individual worms (40 worms per experiment) were transferred to 24-well plates containing 1 ml M9 buffer. After 1 min recovery time, the number of body bends, the reciprocating motion of bends at the mid-body, were scored for 30 s. Mechanosensory neuronal aberrations were measured in the soft-touch sensitivity assay (Pan et al., 2011). On day 5 and day 10 of adulthood, 20–25 randomly selected individuals/experiments were subjected to touch response by counting the number of positive responses to five alternating touches at the posterior and anterior ends of each worm using eyelash (10 total touches/worm). For reproduction assay, worms treated with and without santalol isomers (10 worms per experiment) were individually shifted to fresh NGM plates each day until their reproduction period becomes ceased. The resulting progeny was allowed to develop at 20°C and counted at their adulthood stage. The growth of the worms was measured by body length, which was determined by measuring the flat surface area of control and treated animals (50 worms per experiment) using an image analyzer (Optika, Italy) (Devagi et al., 2018; Mohankumar et al., 2019a). Chemotaxis and food sensing behavior assays were performed as described. For detailed procedures, see the supplementary material.

### Visualization of TTR Aggregation

TTR aggregation was measured by using the transgenic lines expressing several types of TTR::EGFP in body wall muscle cells. Synchronized worms (L1 stage; 10–15 individuals per experiment) were grown on NGM plates with and without santalol isomers. On day 2 and day 6 of adulthood, the worms were collected, washed with M9 buffer, and mounted on glass slides for fluorescent microscopy (BX41, Olympus, Japan). Worms were anesthetized using NaN_3_ at a final concentration of 25 mM. Fluorescence images were obtained, and the visible TTR::EGFP aggregates were counted. The TTR::EGFP aggregates are spherical-shaped discrete structures, and their boundaries are clearly differentiated from nearby fluorescent signals (Tsuda et al., 2018). At least three independent biological trials were performed at similar conditions with appropriate replicates, as indicated.

### Measurement of Intracellular ROS Levels

Intracellular ROS levels in *C. elegans* were measured using the molecular probe H_2_DCF-DA. The worms were maintained and treated as described in the lifespan assay. After treatment, the worms were washed off from the plates with PBST buffer (PBS + 1% Tween 20) and pelleted by centrifugation. The worms were quickly frozen in liquid nitrogen and immediately sonicated for 5 min to disrupt the outer cuticle. The homogenized samples were then transferred to 96-well plates and incubated with 50 μM H_2_DCF-DA solution for 30 min in the dark at 20°C. Fluorescence intensity was measured using a microplate reader at an excitation/emission wavelength of 490/530 nm.

### RNA Interferences

The regular feeding RNAi method was performed to inactivate specified genes, as previously described (Timmons et al., 2001). Individual colonies of *E. coli* strain HT115(DE3) transformed with either the RNAi-encoding plasmid (test) or the empty pL4440 vector (control) were grown in LB media containing 100 μg ml^−1^ ampicillin for 12 h at 37°C. Bacteria were concentrated fivefold and seeded onto NGM plates containing 100 μg ml^−1^ ampicillin and 1 mM IPTG, hood-dried and cultured for 12 h to induce the expression of dsRNA. Synchronized L1 stage worms were transferred to RNAi plates and raised until the young adult stage for efficient knockdowns.

### Quantitative Real-Time PCR for Gene Expression

The worms were frozen in liquid nitrogen, thawed on ice, and homogenized at 4°C. Total RNA was extracted using TRIzol reagents (Invitrogen) (He, 2011) and was reverse transcribed into cDNA using a RevertAid First-strand cDNA synthesis kit (Thermo Scientific™) according to the manufacturer’s protocol. Quantitative real-time PCR was performed with SYBR Green master mix, and relative gene expression was analyzed using a comparative C_t_ (ΔΔC_t_) method. The housekeeping gene *act-1* was chosen as the reference gene.

### Molecular Dynamics Simulation

The molecular docking was implemented in AutoDock Vina (v.1.1.2) to identify the binding affinity of ligands α- and β-santalol towards the TTR_WT_ (PDB ID: **4D7B**) and TTR_V30M_ (PDB ID: **4TL4**) (Trott and Olson, 2009). The molecular dynamics simulation of apoproteins and ligand-bound complexes was performed in the GROMACS v2016.4 package using the CHARMM36 all-atom force field at neutral pH and 310 K. The binding energy components ΔE_vdw_, ΔE_ele_, ΔG_pb_, ΔG_sa_, and ΔG_bind_ were calculated by the molecular mechanics/Poisson-Boltzmann surface area approach. A detailed experimental procedure is given in the electronic supplementary material.

### Statistical Analyses

The lifespan of worms was graphed as Kaplan-Meier survival curves with MedCalc statistical tool (MedCalc 14.8.1, Ostend, Belgium) and analyzed using the log-rank (Mantel-Cox) test. The differences between control and santalol isomers-treated groups were statistically compared by one-way analysis of variance (ANOVA; 95% confidence interval) followed by Bonferroni’s post hoc test (SPSS 17, IBM Corporation, NY, United States) and presented as mean ± standard deviation (SD)/standard error of the mean (SEM) of at least three separate experiments, unless otherwise noted. The probability level of *p* < 0.05 was considered to be statistically significant between the means.

## Results and Discussion

### α- and β-Santalol Inhibit TTR Aggregation and Stabilize the TTR Dimer-Dimer Interface

**FIGURE 1:**
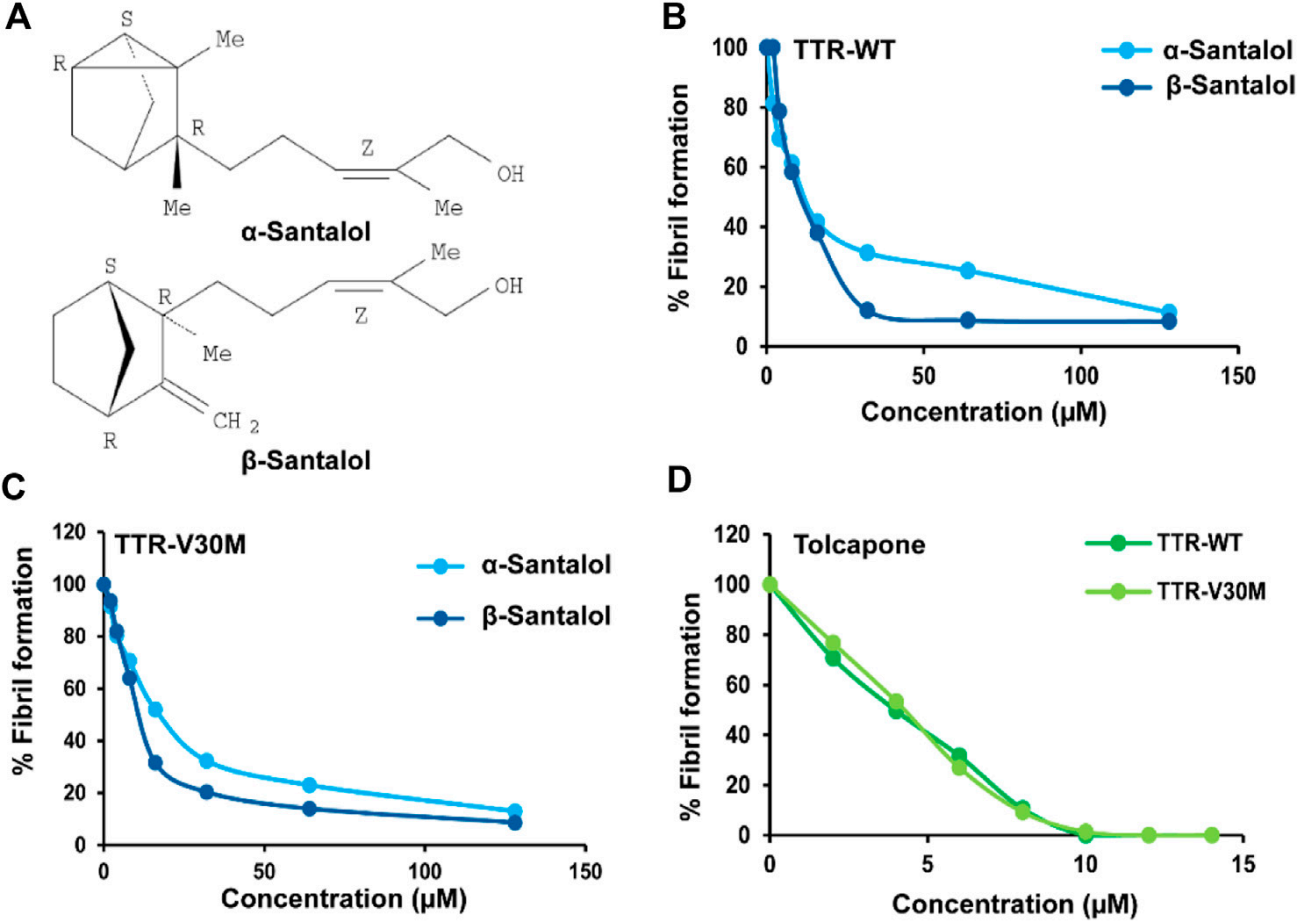
Santalol isomers effects over TTR_WT_ and TTR_V30M_ aggregation. **(A)** Chemical structure of tricyclic α- and β-santalol extracted from *Santalum album* L., oil. Anti-amyloidogenic effect of **(B,C)** santalol isomers and **(D)** tolcapone (positive control) on TTR_WT_ and TTR_V30M_. TTR solutions were incubated with multiple concentrations of santalol isomers or tolcapone, and aggregation was induced by acidification (pH 4.2; 72 h). Turbidity was measured at 340 nm, and the percentage of TTR aggregation was calculated. Combined data from four independent biological trials were presented, each performed in triplicate.

We first tested the TTR inhibitory potential of santalol isomers (Figure 1A) under *in vitro* conditions at low pH (4.2). This acidic pH induces TTR fibril formation, thus increasing sample turbidity and light scattering. The results showed that α- and β-santalol were effective at inhibiting both TTR_WT_ (EC_50_: 36.7 ± 1.2 µM; 31.1 ± 2.4 µM) and TTR_V30M_ aggregation (EC_50_: 44.3 ± 3.5 µM; 34.5 ± 2.8 µM) under acidic denaturation conditions (pH 4.2, 72 h) (Figures 1 B,C). Under similar conditions, the positive control tolcapone more effectively prevents the aggregation of TTR_WT_ and TTR_V30M_ with EC_50_ values of 4.6 ± 0.5 and 4.8 ± 0.7 µM, respectively (Figure 1D, Supplementary Table S1), as measured by light scattering. Next, we addressed whether the anti-aggregational activity of santalol isomers is mediated through tetramer stabilization. Tetramer dissociation is believed to be the rate-limiting step for FAP, and stabilization of the native TTR tetramer through pharmacological interventions is a promising strategy for treating TTR amyloidosis. Several small molecules, nonsteroidal anti-inflammatory drugs, and bioactive phytomolecules have recently been demonstrated to delay or even halt TTR aggregation by kinetically stabilizing the native tetrameric form of TTR by binding to the T_4_-binding sites, thus increasing the energy barrier of tetramer dissociation (Klabunde et al., 2000; Johnson et al., 2005; Sant’Anna et al., 2016; Tsuda et al., 2018). However, some details of this mechanism remain to be explored. The pharmacological stabilization of TTR tetramer with tafamidis has been approved by the European Medicines Agency to treat early-stage FAP and is currently available in Japan and Europe (Bulawa et al., 2012). Unfortunately, tafamidis fails to treat advanced TTR amyloidosis and has some adverse effects (Lozeron et al., 2013; Huber et al., 2019). With this understanding, we performed *in vitro* TTR stabilization assay to address whether the TTR inhibitory potential of santalol isomers was mediated through tetramer stabilization. TTR dissociation was induced by urea and tracked by monitoring the changes in Trp fluorescence (Hurshman et al., 2004). The results showed that α- and β-santalol prevents urea-induced TTR_WT_ and TTR_V30M_ denaturation and allows a large amount of TTR to remain in its native state. It was found that the TTR stabilizing efficacy of santalol isomers was found to be dose-dependent. α- and β-Santalol exerted a strong stabilizing effect on TTR_WT_ and TTR_V30M_ at 64 µM and allowed less than 30% of TTR in the unfolded state upon incubation in 8 M urea, which indicates a strong impact on TTR kinetic stability. In contrast, the percentage of folded TTR was reduced to 61.6% (TTR_WT_) and 75.9% (TTR_V30M_) in the absence of santalol isomers or tolcapone. In the positive control (tolcapone, 8 µM), however, more than 90% of TTR remains in the native state (Supplementary Figure S1).

To further strengthen the *in vitro* results, we, therefore, performed molecular dynamics simulations to identify the possible interaction of santalol isomers with T_4_ binding sites. The crystal structures of tolcapone bound to TTR variants (TTR_WT_ and V122I) were solved previously (Sant’Anna et al., 2016). X-ray crystallography studies have shown that tolcapone buried deep within the T_4_ binding cavity and its 4-methylphenyl ring forms hydrophobic contacts with residues ALA108, LEU110, SER117, and THR119. Additionally, a hydrogen bonding was also observed between tolcapone’s central carbonyl group and the hydroxyl side chain of THR119. However, the atomic insights on the binding of santalol isomers to TTR remain unknown. Therefore, the crystal structures of TTR_WT_ and TTR_V30M_ were subjected to 50 ns MD simulation at physiological conditions in a neutral TIP3P aqueous box employing the CHARMM36 all-atom force field. The final stable apoprotein structures at 50 ns were extracted and used as receptors for molecular docking analysis. The active site of the TTR variants was predicted using CASTp (Tian et al., 2018), and ligands α- and β-santalol were docked to the predicted binding site. To study subsequent conformational changes on TTR_WT_ and TTR_V30M_ complexes upon ligand binding, the best poses selected from docking analysis were subjected to further 50 ns MD simulation at physiological conditions in a neutral TIP3P aqueous box employing the CHARMM36 all-atom force field. For comparison purposes, the apo forms of TTR_WT_ and TTR_V30M_ were also simulated. The apo TTR_WT_ and TTR_V30M_ reached an equilibrium around 20 ns. Thus, we decided the simulation duration of 50 ns was sufficient to analyze the conformational changes and intermolecular interactions.

α-Santalol showed a binding affinity of −5.9 kcal/mol towards TTR_WT_; it is observed that residues LEU110 (2.84 Å), SER117 (2.49 Å), and THR118 (3.31 Å) were involved in hydrogen bonding, whereas residues ALA108 (3.80 Å) and LEU110 (3.84 Å) were hydrophobic contacts. β-Santalol showed a binding affinity of −5.5 kcal/mol towards the TTR_WT_ and formed five hydrogen bonds with residues ALA108 (2.36 Å), LEU110 (2.85 Å), SER117 (2.48 Å), THR118 (3.29 Å), whereas the residues THR119 (3.34 Å), and LYS15 (3.53 Å), LEU17 (3.71 Å), ALA108 (3.60 Å), and LEU110 (3.42 Å) were hydrophobic contacts. The binding affinity of α- and β-santalol towards the TTR_V30M_ binding site were −5.4 and −6.1 kcal/mol, respectively. α-Santalol interacts with the residue THR119 (2.71 Å) through hydrogen bonding and has strong hydrophobic interactions with residues VAL93 (3.67 Å), PHE95 (3.58 Å), PHE95 (3.70 Å), THR118 (3.74 Å), ALA120 (3.62 Å), and VAL122 (3.48 Å). β-Santalol forms two hydrogen bonds with residue THR96 (2.16 Å, 2.18 Å), and residues PHE95 (3.40 Å), THR105 (3.59 Å), THR118 (3.65 Å), and ALA120 (3.31 Å) were hydrophobic contacts. Hydrogen bonding and hydrophobic contacts play a crucial role in forming and stabilizing energetically-favored protein-ligand complexes (Patil et al., 2010; Sivaramakrishnan et al., 2019). The post docking analysis suggests that the santalol isomers possess a higher binding affinity towards TTR_WT_ than TTR_V30M_.

**FIGURE 2:**
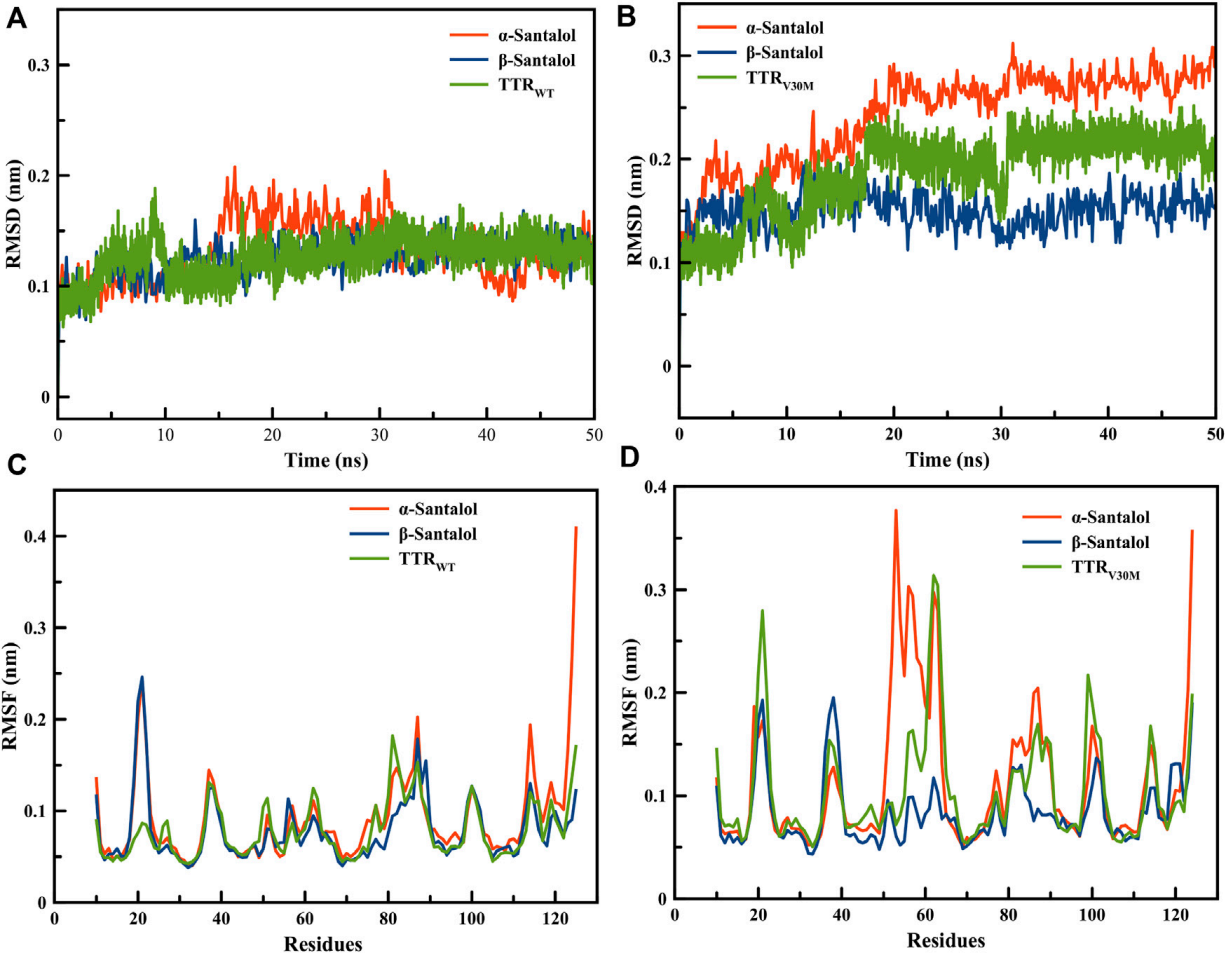
Root mean square deviations for backbone atomic position of **(A)** TTR_WT_ and **(B)** TTR_V30M_ in complex with santalol isomers throughout 50 ns MD simulations. Root mean square fluctuation during the MD simulation run for the critical amino acids of **(C)** TTR_WT_ and **(D)** TTR_V30M_ in complex with santalol isomers.

TTR_WT_ and TTR_V30M_ apo forms showed RMSD values of 0.2 and 0.15 Å, respectively. RMSD curves of both apo forms and ligand-bound complexes were less than 0.3 Å during the 20 ns simulation; even the most flexible complexes cannot touch 0.3 Å. This implies that all the structures reached the equilibrium state during the simulation. β-Santalol has a larger RMSD for the TTR_V30M_ than that of TTR_WT_. As shown in Figures 2A,B, β-santalol bounded TTR variants obtained high stability after 20 ns, whereas α-santalol bounded complexes exhibited noticeable fluctuations. In the case of TTR_V30M_, RMSD for α-santalol bounded complexes increases throughout the simulation, while for TTR_WT_, a spike in RMSD values was initially noted, decreased for a short duration, and then attained equilibrium at 40 ns. As shown in Figures 2A,B, β-santalol bounded TTR variants exhibited enhanced conformational stability than the α-santalol bounded TTR variants during the 20–50 ns.

The RMSF analysis was performed to estimate the fluctuation of each residue on TTR variants upon ligand binding. As presented in Figures 2C,D, the RMSF plot shows a variation in the degree of flexibility among residues in both apo form and ligand bounded TTR complexes, indicating that the loop regions showed more flexibility than β-sheets. Moreover, the TTR with point mutation V30M results in a higher degree of flexibility than the wild-type, especially in the active site and homotetramer interface regions. β-Santalol is bound to both TTR_WT_ and TTR_V30M_ and reduces the flexibility at the residue level. In both TTR_WT_ and TTR_V30M_ apo forms, a large magnitude fluctuation was observed in the homotetramer interface until the binding of β-santalol. The RMSD and RMSF analysis of MD trajectories suggest binding of β-santalol reduces the flexibility of the homotetramer interface and provides enhanced conformational stability to the TTR variants.

To evaluate the energetics of binding in a more reliable and elaborate way, the energy components ΔE_vdw_, ΔE_ele_, ΔG_pb_, ΔG_sa_, and ΔG_bind_ were calculated for ligand-bound complexes by the molecular mechanics/Poisson-Boltzmann surface area approach. As sown in Table 1, the van der Waals energy (ΔE_vdw_) of the complexes made the most contribution to the total binding free energy; a similar result was previously observed (Dong et al., 2016). It is clear that both santalol isomers form energetically-favored complexes with TTR variants; however, ΔG_bind_ values suggest that β-santalol has slightly stronger binding towards TTR_WT_ and TTR_V30M_. These results are highly consistent with molecular docking analysis and MD simulations. From these observations, it was concluded that the santalol isomers interact with TTR variants and reduce the flexibility of the T_4_ binding cavity and the homotetramer interface, which in turn confers conformational stability for TTR variants and prevents the dissociation of the tetramer and subsequent self-assembly into toxic oligomers and aggregates.

**TABLE 1:** ΔE_vdw_, ΔE_ele_, ΔG_pb_, and ΔG_sa_ were calculated by the molecular mechanics/Poisson-Boltzmann surface area approach for TTR_WT_ and TTR_V30M_ proteins in complex with santalol isomers along with the corresponding binding free energies.

Complexes	ΔE/(kcal mol^−1^)				
	ΔE_vdw_	ΔE_ele_	ΔG_pb_	ΔG_sa_	ΔG_bind_
α-Santalol_TTR_WT_	−43.231	−1.782	20.907	−6.164	−30.270
β-Santalol_TTR_WT_	−48.082	−0.892	14.984	−7.034	−41.024
α-Santalol_TTR_V30M_	−37.662	−2.200	27.893	−5.639	−17.608
β-Santalol_TTR_V30M_	−32.972	−0.906	15.302	−4.889	−23.465

### α- and β-Santalol Synergize to Reduce Wild-Type and Val30Met Mutant TTR Aggregates *In Vivo*

**FIGURE 3:**
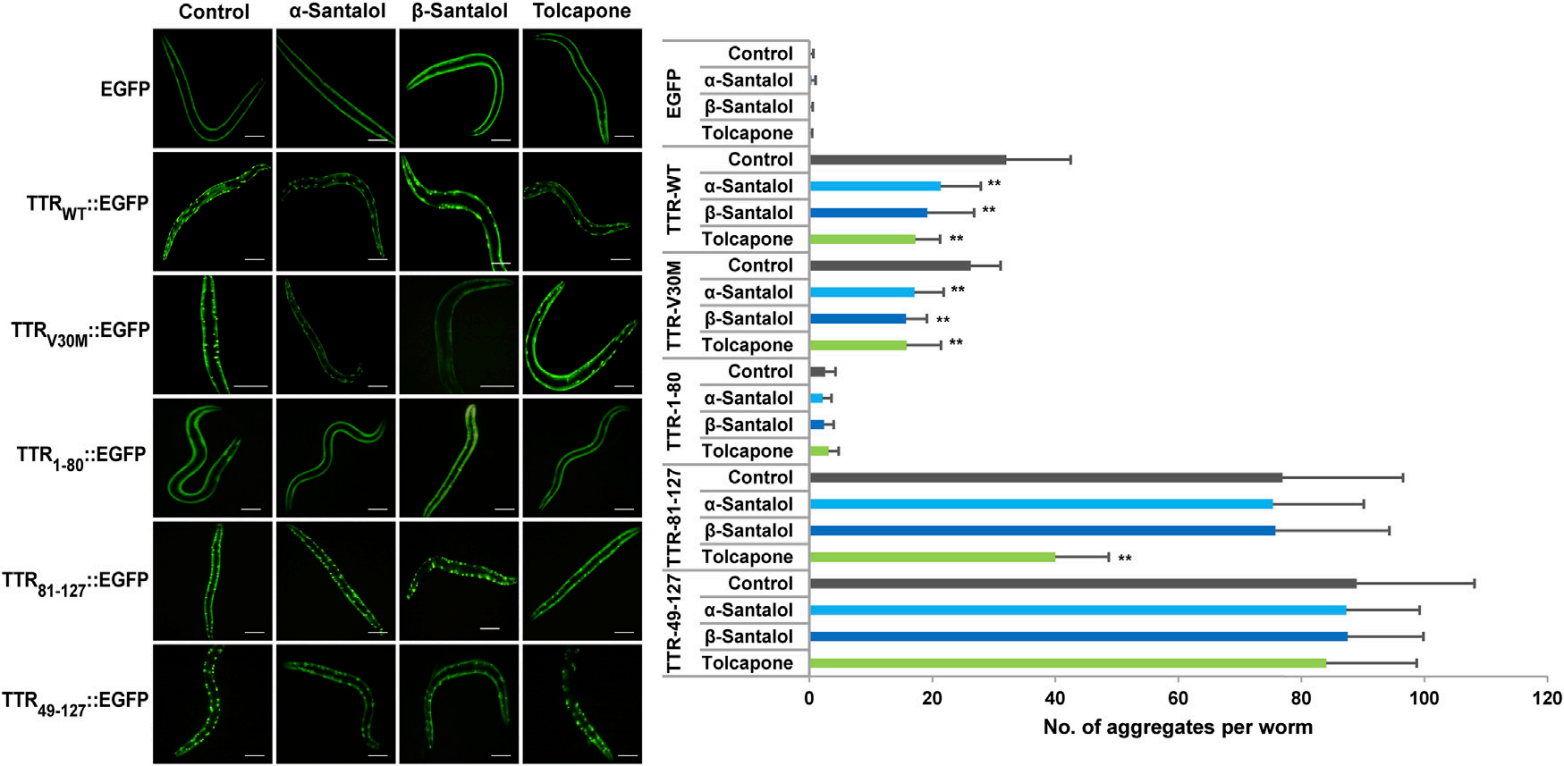
Inhibitory effect of santalol isomers on the formation of TTR aggregates in day 6 adulthood worms. *C. elegans* strains expressing EGFP, TTR_WT_::EGFP, TTR_V30M_::EGFP, TTR_1–80_::EGFP, TTR_81–127_::EGFP, and TTR_49–127_:EGFP were used. The scale bar represents 100 μm. Data are presented as mean ± SD; **p < 0.01 vs. control. See Supplementary Table S2 for more detailed statistics.

To further examine the effect of santalol isomers on the formation of TTR aggregates *in vivo*, novel transgenic strains expressing several types of fragments and full-length TTR was used. In ATTR amyloidosis, amyloid deposits in tissues consist of full-length TTR and C-terminal TTR fragments. C-terminal fragments of TTR play an important role in aggregate formation *in vitro* as well as *in vivo* (Saelices et al., 2015; Tsuda et al., 2018). Therefore, to investigate the inhibitory effect of santalol isomers against TTR aggregation *in vivo*, *C. elegans* models expressing full-length and C-terminal TTR fragments fused with an enhanced green fluorescent protein (EGFP) in body wall muscle cells *viz*., TTR_WT_::EGFP (the full-length wild-type TTR), TTR_V30M_::EGFP (the full-length TTR but containing V30M mutation), TTR_1–80_::EGFP (the 1–80 residue fragment), TTR_81–127_::EGFP (the 81–127 resides fragment), TTR_49–127_::EGFP (the 49–127 residue fragment), and EGFP alone (as control) were used (Tsuda et al., 2018). TTR_WT_ and TTR_V30M_ are the most common TTR variants, TTR_81–127_ contains two strong amyloidogenic β-strands—F and H, and TTR_49–127_ was previously identified in patients with ATTR amyloidosis (Bergström et al., 2005). Moreover, the morphological and structural heterogeneity of the TTR fibrils determines the degree of deposition and reflects the fundamental differences in the pathogenesis of TTR-associated amyloidosis. As a result, we found that worms bearing EGFP alone and TTR_1–80_::EGFP displayed a diffuse localization pattern throughout the body wall muscle cells. TTR_WT_::EGFP and TTR_V30M_::EGFP worms gradually developed aggregates with age, and TTR_49–127_::EGFP and TTR_81–127_::EGFP worms had spherical shaped discrete aggregates (Figure 3; Table 2; Supplementary Figure S2).

**TABLE 2:** Synergistic effect of santalol isomers on the aggregation of different TTR::EGFP aggregates.

Genotype	Treatment (α-+β-santalol)	No. of TTR aggregates per worm (*n* = 40)			
		Day 2		Day 6	
		No. of aggregates ±SD	% Change	No. of aggregates ±SD	% Change
TTR_WT_::EGFP	Control	7.73 ± 3.62		32.03 ± 10.44	
	Tolcapone	5.33 ± 3.78 ns	(–) 31.03	17.23 ± 4.07**	(–) 46.20
	4 + 4 µM	7.83 ± 3.92 ns	(+) 1.29	31.47 ± 7.84*	(–) 1.77
	4 + 8 µM	7.67 ± 4.33 ns	(–) 0.86	25.43 ± 8.92 ns	(–) 20.60
	4 + 16 µM	7.17 ± 3.53 ns	(–) 7.33	28.40 ± 10.75 ns	(–) 11.34
	8 + 4 µM	7.83 ± 4.94 ns	(+) 1.29	31.91 ± 6.08 ns	(–) 0.21
	8 + 8 µM	7.63 ± 5.07 ns	(–) 1.29	31.30 ± 7.80**	(–) 2.29
	8 + 16 µM	7.20 ± 3.66 ns	(–) 6.90	24.33 ± 9.89**	(–) 24.04
	16 + 4 µM	7.83 ± 4.40 ns	(+) 1.29	23.00 ± 5.58**	(–) 28.20
	16 + 8 µM	4.80 ± 2.96 ns	(–) 37.93	11.87 ± 5.12**	(–) 62.96
	16 + 16 µM	7.23 ± 4.08 ns	(–) 6.47	22.93 ± 5.00**	(–) 28.41
	32 + 4 µM	7.20 ± 3.68 ns	(–) 6.90	22.03 ± 5.36**	(–) 31.22
	32 + 8 µM	7.07 ± 2.80 ns	(–) 8.62	21.40 ± 4.82**	(–) 33.19
	32 + 16 µM	6.47 ± 4.25 ns	(–) 16.38	17.63 ± 5.35**	(–) 44.95
TTR_V30M_::EGFP	Control	8.13 ± 4.30		26.23 ± 4.88	
	Tolcapone	6.17 ± 2.76 ns	(–) 24.18	15.83 ± 5.56**	(–) 39.64
	4 + 4 µM	8.30 ± 3.64 ns	(+) 2.05	27.07 ± 6.64 ns	(+) 3.18
	4 + 8 µM	8.40 ± 4.45 ns	(+) 3.28	25.07 ± 8.91 ns	(–) 4.45
	4 + 16 µM	8.00 ± 3.93 ns	(–) 1.64	24.10 ± 7.55 ns	(–) 8.13
	8 + 4 µM	7.77 ± 3.56 ns	(–) 4.51	21.10 ± 7.91 ns	(–) 19.57
	8 + 8 µM	7.60 ± 3.41 ns	(–) 6.56	22.57 ± 7.61 ns	(–) 13.98
	8 + 16 µM	7.77 ± 3.46 ns	(–) 4.51	21.10 ± 9.60 ns	(–) 19.57
	16 + 4 µM	7.30 ± 3.41 ns	(–) 10.25	20.73 ± 6.73 ns	(–) 20.97
	16 + 8 µM	5.17 ± 2.78 ns	(–) 36.48	12.47 ± 4.28**	(–) 52.48
	16 + 16 µM	7.63 ± 4.51 ns	(–) 6.15	21.23 ± 6.31 ns	(–) 19.06
	32 + 4 µM	7.17 ± 3.09 ns	(–) 11.89	20.13 ± 6.32 ns	(–) 23.25
	32 + 8 µM	7.63 ± 4.22 ns	(–) 6.15	20.60 ± 6.41 ns	(–) 21.47
	32 + 16 µM	7.17 ± 3.74 ns	(–) 11.89	18.97 ± 6.43**	(–) 27.70

Data are presented as mean ± SD; ns-not significant, *p < 0.05, and **p < 0.01 vs. control.

In this study, tolcapone was used as a positive control and is an orally active and reversible inhibitor of catechol-*O*-methyltransferase (COMT; EC 2.1.1.6), which is approved in Europe and the United States for the treatment of Parkinson’s disease. It has been shown to prevent the onset of TTR misfolding and TTR-induced toxicity by specifically binding and stabilizing the native tetrameric structure of TTR more effectively than clinically approved drugs for early-stage FAP, diflunisal and tafamidis (Sant’Anna et al., 2016). To measure the effect of these drug candidates on the formation of TTR aggregates, we counted the number of spherical shaped discrete structures in the transgenic worms grown in the presence or absence of α-santalol (32 µM), β-santalol (16 µM), and tolcapone (6 µM) at 20°C. As shown in Figure 3, the results showed that supplementation of santalol isomers significantly inhibited the formation of TTR aggregates in worms expressing TTR_WT_::EGFP and TTR_V30M_::EGFP across various adulthood stages (day 2 and day 6). However, α- and β-santalol treatment exhibited a negligible effect on the aggregation of TTR_1–80_::EGFP, TTR_81–127_::EGFP, and TTR_49–127_::EGFP when compared to that of untreated control. It was interesting to note that the TTR inhibitory potential of santalol isomers was found to be gradually increase as the worms aged. In earlier developmental stage (day 2 adulthood), α- and β-santalol treatment marginally decreased TTR_WT_ [21.6% (*p* = 0.44) and 19.8% (*p* = 0.59)] and TTR_V30M_ [16.8% (*p* = 0.84) and 29.1% (*p* = 0.0.06)] aggregates (Supplementary Figure S2). In addition, santalol isomers treatment was highly effective at inhibiting both TTR_WT_ [33.3% (*p* < 0.01) and 40.2% (*p* < 0.01)] and TTR_V30M_ [34.8% (*p* < 0.01) and 40.2%; (*p* < 0.01)] aggregation in day 6 adulthood worms. The effect of santalol isomers in preventing TTR aggregation was on par with the positive control tolcapone. Tolcapone treatment inhibits the aggregate formation in TTR_WT_::EGFP [day 2–31.0% (*p* = 0.06), day 6–46.2% (*p* < 0.01)], TTR_V30M_::EGFP [day 2–24.2% (*p* = 0.21), day 6–39.6% (*p* < 0.01)], and TTR_81–127_::EGFP [day 2–30.3% (*p* < 0.01), day 6–48.0% (*p* < 0.01)] worms; however, it did not significantly affect TTR_1–80_::EGFP, TTR_49–127_::EGFP aggregates, but tolcapone treatment slightly reduce TTR formation (*p* > 0.05). We also explored the efficacy of combining α- and β-santalol on the aggregation of TTR in *C. elegans*. We systematically evaluated 12 possible combinations that can be formed out of four doses of α-santalol (4, 8, 16, and 32 µM) and three doses of β-santalol (4, 8, and 16 µM). It was observed that α-santalol combined with β-santalol, each at its half-optimal dose (16 and 8 μM, respectively), lead to a significant reduction in the TTR_WT_::EGFP [day 2–37.9% (*p* = 0.37); day 6–63.0% (*p* < 0.01)] and TTR_V30M_::EGFP [day 2–36.5% (*p* = 0.19); day 6–52.5% (*p* < 0.01)] aggregates compared to the groups treated with single molecule and the control group. A few of the tested combinations also produced a noticeable impact on TTR aggregation, while their effect was not found to be synergistic than the half-optimal dose of santalol isomers, and some combinations showed no benefits (Table 2). These results indicated that the synergism between α- and β-santalol has the potential to inhibit the onset of TTR misfolding and aggregate formation in *C. elegans*.

### α- and β-Santalol Extend the Lifespan of TTR Expressing *C. elegans*

**FIGURE 4:**
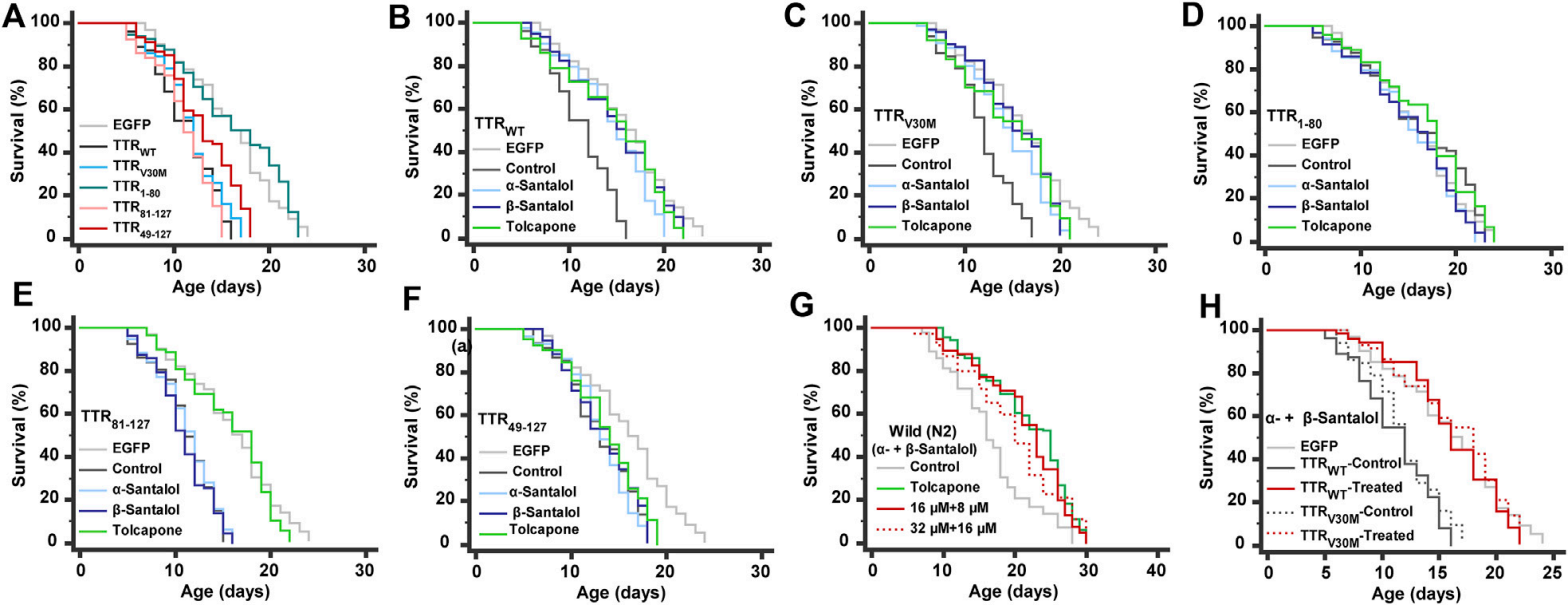
Lifespan analyses. **(A)** The lifespan of worms expressing various TTR fragments. **(B**–**F)** Effect of α- and β-santalol on the lifespan of TTR protein-expressing worms. Synergistic effect of santalol isomers on the lifespan of **(G)** wild-type and **(H)** TTR transgenic worms. Combined data of three independent biological trials were presented. See Supplementary Tables S3, S4 for statistical details of lifespan analyses.

Patients with TTR amyloidosis demonstrate several pathophysiological changes that ultimately lead to death within 10 years (Ando et al., 2005). Therefore, a small molecule with antiaging properties that can also improve TTR degradation could be a viable strategy to treat FAP. We have previously shown that santalol isomers reduced the aggregation of toxic amyloid-β, α-synuclein, and polyglutamine proteins in *C. elegans*; these proteins are involved in the key pathological events in Alzheimer’s, Parkinson’s, and Huntington’s diseases, respectively. In addition, santalol isomers extend the lifespan of *C. elegans* strains expressing these toxic proteins in body wall muscle cells and neurons (Mohankumar et al., 2018; Mohankumar et al., 2019b; Mohankumar et al., 2020). Taking into account the anti-aggregation and anti-aging potential of santalol isomers, we have tested their effects on the lifespan of worms expressing several TTR aggregates. Under standard laboratory conditions, the lifespan of worms expressing EGFP (control; 15.9 ± 0.4 days, *p* = 0.39) and TTR_1–80_ (16.3 ± 0.5 days, *p* = 0.23) did not obviously alter compared to their wild-type counterparts (16.1 ± 0.5 days). However, worms expressing TTR_WT_ (29.3%, *p* < 0.0001), TTR_V30M_ (25.3%, *p* < 0.0001), TTR_81–127_ (30.3%, *p* < 0.0001), and TTR_49–127_ (17.8%, *p* < 0.0001) fragments had a significantly shorter lifespan (Figure 4A), suggest that these TTR fragments are likely proteotoxic, as is believed to be the case in humans. These findings are highly consistent with the previous reports (Madhivanan et al., 2018; Tsuda et al., 2018). We found that continuous exposure to α- and β-santalol treatment initiated during development (beginning from an embryo) led to lifespan extension in worms expressing TTR. After administering 32 µM α-santalol, the mean lifespan of TTR_WT_ and TTR_V30M_ *C. elegans* increased to 14.4 ± 0.4 and 14.4 ± 0.3 days, respectively, which expanded the lifespan of worms by 27.6% (*p* < 0.0001) and 20.8% (*p* < 0.0001), respectively, compared to the control groups (Figures 4B,C). Similarly, β-santalol (16 µM) treatment also significantly increased the mean lifespan of TTR_WT_ (Figure 4B) and TTR_V30M_ (Figure 4C) worms by 32.9% (*p* < 0.0001) and 27.8% (*p* < 0.0001), respectively. However, α- and β-santalol treatment would not further extend the lifespan of worms expressing TTR_1–80_ (*p* = 0.0958; *p* = 0.1264) (Figure 4D), TTR_81–127_ (*p* = 0.5076; *p* = 0.5074) (Figure 4E), and TTR_49–127_ (*p* = 0.3274; *p* = 0.6365) (Figure 4F). We next investigated whether α-santalol and β-santalol can synergize to reduce the TTR toxicity and enhance the lifespan of TTR expressing *C. elegans*. To test this idea, initially we measured the lifespan of wild-type worms cultured on NGM plates carrying different concentrations of santalol isomers to identify the synergistic dose(s). Firstly, we observed that 16 µM α-santalol and 8 µM β-santalol together were the minimal dose to synergistically extend the lifespan of wild-type *C. elegans* by 28.0% under standard laboratory conditions (Figure 4G; Supplementary Figure S3). The combinatorial effect of santalol isomers is significantly higher (22.3%, *p* < 0.01) than the largest effect produced by each compound under similar conditions (Mohankumar et al., 2018; Mohankumar et al., 2020). Interestingly, the combination of α- and β-santalol at their optimal doses (32 and 16 μM, respectively) is not synergistic and extends the mean lifespan of N2 worms by 19.0% (Figure 4G; Supplementary Figure S3). Secondly, we found that the dual combination of santalol isomers (16 µM α-santalol+8 µM β-santalol) consistently extended the mean lifespan of TTR_WT_ and TTR_V30M_ expressing worms by 43.0 and 37.3%, while it did not obviously alter the lifespan of TTR_1–80_ (*p* = 0.0826), TTR_81–127_ (*p* = 0.2477), and TTR_49–127_ (*p* = 0.2497) worms (Figure 4H). In contrast to the dual combination, both α- and β-santalol were less effective separately on TTR worms. From this observation, it was apparent that a one-fold lower dose of santalol isomers together had a better pro-longevity effect on *C. elegans*; therefore, for the remainder of this study, we focused on the dual combination.

**FIGURE 5:**
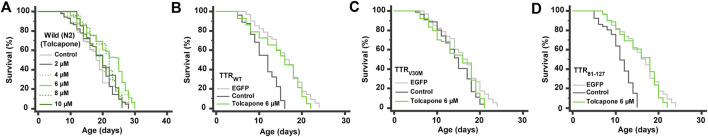
Lifespan analyses. Life promoting ability of tolcapone (positive control) on **(A)** wild-type, **(B)** TTR_WT_, **(C)** TTR_V30M_, and **(D)** TTR_81-127_ worms. Combined data of three independent biological trials were presented. See Supplementary Table S3 for statistical details of lifespan analyses.

Surprisingly, we found that different pharmacological doses of tolcapone had different performances. Of the five concentrations, the survival curve of wild-type worms treated with 6 µM tolcapone manifested not only a significant shift to the right but also had a mean lifespan of 21.9 ± 0.5 days (18.1 ± 0.5 days, untreated control) with a maximum lifespan of 30 days and increased by 21.2% in mean lifespan (*p* < 0.0001) (Figure 5A). Worms treated with 2 and 4 µM tolcapone did not show a significant increase in mean lifespan, but their treatment marginally increased the mean lifespan by 2.3% (*p* = 0.5247) and 5.5% (*p* = 0.0310), respectively. Furthermore, worms cultured on 8 and 10 µM tolcapone displayed a significantly different right-shifted survival curve when compared with unexposed control, and the mean lifespan was increased by 13.8 and 7.8%, respectively. Therefore, 6 µM tolcapone was selected as the optimal concentration for most subsequent studies. On the other hand, tolcapone feeding increased the mean lifespan of TTR_WT_ (Figure 5B), TTR_V30M_ (Figure 5C), and TTR_81–127_ (Figure 5D) worms by 31.2% (*p* < 0.0001), 23.0% (*p* < 0.0001), and 41.7% (*p* < 0.0001) respectively, and it would not further extend the lifespan of TTR_1–80_ (3.3%, *p* = 0.4205) and TTR_49–127_ (4.0%, *p* = 0.0906) worms. Together these data imply that tolcapone can extend the lifespan of wild-type worms and worms expressing TTR at a relatively low dose; this may reduce or minimize the potential side effects (Olanow, 2000), which is relevant for future therapy against TTR amyloidosis. Our data provided the first insight into the life-promoting properties of tolcapone, which will further promote it as an antiaging drug for a longer and healthier life for patients with TTR and related amyloidosis. However, the precise mechanism(s) underlying the longevity-promoting properties of tolcapone is still unexplored, and we are currently testing these possibilities.

## Synergistic Inhibition of Transthyretin by Santalol Isomers Requires SKN-1/Nrf2, Autophagy, and Proteasome Functions

**FIGURE 6:**
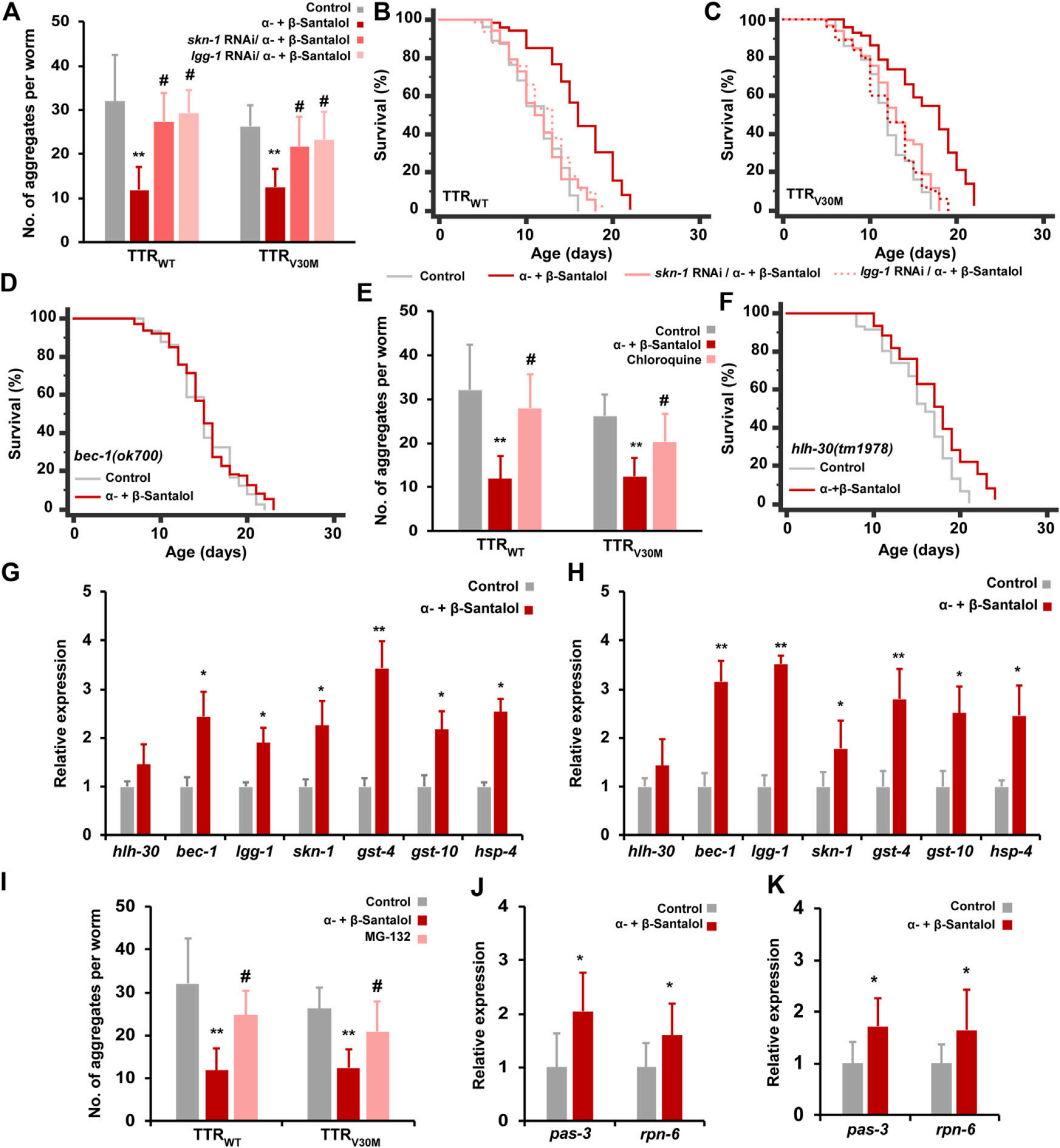
The beneficial synergistic effect of santalol isomers on TTR aggregation requires SKN-1, autophagy, and proteasome. **(A)** Levels of TTR aggregates in worms treated with α-+β-santalol fed-with *skn-1* and *lgg-1* RNAi. **(B,C)** Survivorship curves of TTR_WT_ and TTR_V30M_ worms deficient in *skn-1* and *lgg-1* (RNAi). **(D)** Survivorship curves of *bec-1* mutants raised at 20°C on plates containing santalol isomers. **(E)** The blocking effect of chloroquine on TTR_WT_ and TTR_V30M_ aggregates in worms treated with santalol isomers. **(F)** Survivorship curves of *hlh-30* loss-of-function worms treated with santalol isomers. **(G,H)** Relative expression rate of autophagy and SKN-1 target genes in TTR_WT_ and TTR_V30M_ worms. **(I)** The blocking effect of MG-132 on TTR_WT_ and TTR_V30M_ aggregates in worms treated with santalol isomers. **(J,K)** Relative expression rate of proteasome subunits in TTR_WT_ and TTR_V30M_ worms. Data are presented as mean ± SD; *p < 0.05, **p < 0.01 vs. control; #*p* < 0.01 vs. α-+β-santalol. See Supplementary Table S5 for statistical details of lifespan analyses.

We next investigated the mechanism(s) by which the combination of α-+β-santalol extends lifespan and inhibits TTR aggregation in *C. elegans*. In our previous study, we confirmed that α- and β-santalol selectively regulate the SKN-1/Nrf2 protective pathway to reduce oxidative (H_2_O_2_) (Mohankumar et al., 2019b), neurotoxic (6-OHDA) (Mohankumar et al., 2018), and proteotoxic (α-synuclein, β-amyloid, and polyQ) (Mohankumar et al., 2018; Mohankumar et al., 2020) stresses in *C. elegans*. It has been shown before that santalol isomers trigger the activation of autophagy in keratinocytes (Dickinson et al., 2014). Autophagy is a complex lysosome-dependent intracellular machinery that degrades misfolded and abnormal protein aggregates to maintain proteostasis. The accumulation of abnormal protein aggregates, a key pathological event in neurodegenerative diseases, can be reduced by autophagy. Dysregulated autophagic activity has been observed in most neurodegenerative diseases, which accelerates the disease progression (Nixon, 2013). Recent studies have shown that autophagy is impaired in experimental models and patients with TTR amyloidosis (Buxbaum, 2009). In *C. elegans*, autophagic activity has been reported to reduce TTR-mediated neurotoxicity and TTR oligomer and tetramer levels (Madhivanan et al., 2018). Extracellular deposition of mutant TTR fibril has also been shown to downregulate proteasome functions (Santos et al., 2007). The proteasome is a multisubunit proteinase complex that is critical for the degradation of damaged and misfolded cellular proteins by the ubiquitin-proteasome system (UPS). Therefore, activating the SKN-1/Nrf2 protective pathway, autophagy, and the proteasome should be considered therapeutic targets for treating TTR amyloidosis. Having established that the combination of α- and β-santalol inhibits TTR aggregation and prolongs the lifespan of *C. elegans* expressing TTR fragments, we wondered if this effect was indeed due to the activation of SKN-1/Nrf2 and autophagic activity. First, we tested the requirement of SKN-1 in santalol isomers-mediated inhibition of TTR aggregation. Knockdown of *skn-1* using RNAi significantly blocked the reduction in TTR aggregation and prolongation of lifespan by α-+β-santalol in TTR_WT_ (*p* < 0.01) and TTR_V30M_ (*p* < 0.01) worms (Figures 6A–C), suggesting the involvement of SKN-1. We next examined the role of autophagy in santalol isomers-induced reduction in TTR toxicity; therefore, we tested α-+β-santalol effects in TTR_WT_::EGFP and TTR_V30M_::EGFP worms depleted of *lgg-1* (*lgg-1* RNAi) and *bec-1*(*ok700*) (mutant). *lgg-1* and *bec-1* are mammalian homologs of MAP-LC3 and Beclin1, respectively, and are required for the breakdown of cellular components by autophagy. It was observed that animals silenced with *lgg-1* RNAi were unresponsive to α-+β-santalol treatment, since the number of TTR aggregates in TTR_WT_ (*p* = 0.82) and TTR_V30M_ (*p* = 0.27) worms were statistically similar to unexposed groups that received control RNAi or solvent (Figure 6A). *lgg-1* RNAi significantly reversed the longevity-promoting efficacy of α-+β-santalol feeding in worms expressing wild-type (25.5%; *p* < 0.0001) (Figure 6B) and V30M mutant (25.1%; *p* < 0.0001) (Figure 6C) TTR. Furthermore, santalol isomers failed to extend the mean lifespan of worms bearing a loss of function mutation in the *bec-1* gene (*p* = 0.3404), identifying the underlying role of *bec-1* in santalol isomers-mediated TTR inhibition (Figure 6D). To further study the role of autophagy, we blocked the autophagic flux using chloroquine (10 mM). Chloroquine, a well-established antimalarial drug, has previously been shown to inhibit autophagic flux by reducing autophagosome-lysosome fusion and activity (Mauthe et al., 2018). The results showed that chloroquine treatment blocked the reduction in TTR_WT_ and TTR_V30M_ aggregation by α-+β-santalol, so the worms treated with chloroquine/α-+β-santalol had almost the same aggregation pattern as unexposed worms (*p* = 0.16 and *p* = 0.074, respectively) (Figure 6E). These results showed that autophagy is required for the synergistic effect of α-+β-santalol on TTR aggregation.

Given the dependency of autophagy in santalol isomers-mediated TTR inhibition, we tested the involvement of HLH-30 transcription factor. Recently, HLH-30, a mammalian helix-loop-helix transcription factor EB (TFEB), was shown to regulate longevity, stress resilience, and protein aggregation in *C. elegans*. In addition, HLH-30 regulates the expression of several genes involved in activating autophagy and lysosomal functions (Lapierre et al., 2013; Lin et al., 2018). α-+β-Santalol treatment extended the lifespan of worms carrying the *hlh-30*(*tm 1978*) loss-of-function allele by 11.4% (*p* < 0.0001) (Figure 6F), but the increase was not the same as for wild-type N2 worms. We also noticed an increase in *hlh-30* mRNA levels in TTR_WT_ and TTR_V30M_ worms after treatment with α-+β-santalol, but the increment was not statistically significant (Figures 6G,H). These results suggest that santalol isomers partially require the HLH-30 transcription factor to alleviate TTR-associated pathologies. To further validate the results, we measured the expression rate of SKN-1 direct target and autophagy genes in TTR_WT_ and TTR_V30M_ worms. α-+β-Santalol treatment significantly upregulates the expression levels of autophagy genes *bec-1* and *lgg-1* and *skn-1* and its direct transcriptional readouts including *gst-4*, *gst-10* (antioxidant genes), and *hsp-4* (ER chaperon) (Figures 6G,H). We also hypothesized that santalol isomers may have altered the proteasome system in worms expressing TTR fragments. To test the possibility, we first used the proteosome inhibitor MG-132 to block the proteasome functions and to check whether proteasome functions are necessary for the beneficial effect of santalol isomers on TTR aggregation in *C. elegans*. As shown in Figure 6I., 20 µM MG-132 completely reversed the TTR aggregation inhibitory effect of α-+β-santalol in TTR_WT_ and TTR_V30M_ worms. Thus, α-+β-santalol inhibits the proteotoxicity and pathological symptoms of TTR by enhancing proteasome functions. We, therefore, evaluated the mRNA levels of various proteasome subunits (*pas-1-7*; *pbs-1-7*; *rpt-1-6*; *rpn-1-3*, and *rpn-5-12*) in TTR_WT_ and TTR_V30M_ worms treated with santalol isomers. After treatment with α-+β-santalol, the transcript levels of *pas-3* and *rpn-5* were significantly upregulated (*p* < 0.05) (Figures 6J,K; Supplementary Figure S4). PAS-3 (20S proteasome subunit alpha type-4) is one of the critical components of the 20S proteasome and is involved in the proteasomal protein catabolic process (wormbase.org). RPN-5 (proteasome 26S subunit, non-ATPase 12) is a conserved proteasome subunit required for proper proteasome localization and assembly (Yen et al., 2003). Altogether, these results suggested that santalol isomers exert their beneficial effects against TTR aggregation by activating the SKN-1/Nrf2 protective pathway, autophagy, and proteasome in *C. elegans*.

## Santalol Isomers Improved the Physiological Functions of Transthyretin Expressing *C. elegans*

**FIGURE 7:**
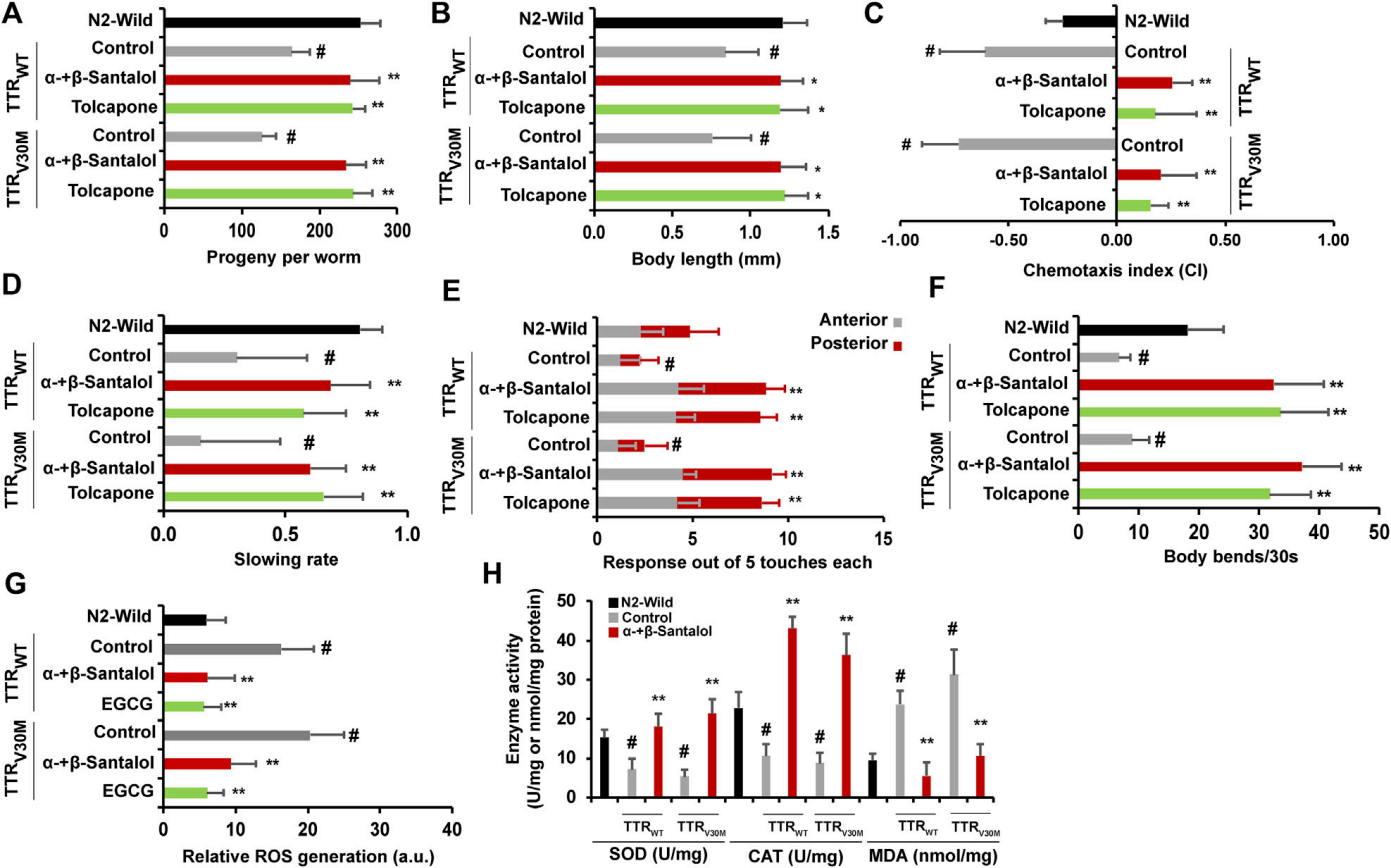
Santalol isomers confer favorable healthspan benefits on *C. elegans* expressing TTR proteins. Synergistic effect of α-+β-santalol on **(A)** reproduction, **(B)** development, **(C)** chemotaxis, **(D)** food sensing, **(E)** touch response, **(F)** body bends, **(G)** ROS levels, and **(H)** antioxidant enzymes and MDA levels in TTR_WT_ and TTR_V30M_ worms. Data are presented as mean ± SD; #*p* < 0.01 vs. wild-type; *p < 0.05, **p < 0.01 vs. control.

Interventions that inhibit the aggregation of toxic proteins and improve life expectancy do not necessarily increase health expectancy with age (Mohankumar et al., 2020). To test this idea, we examined the beneficial effects of α-+β-santalol on a few physiological parameters such as reproduction (progeny production), development (body length), chemotaxis, food sensing, motor activity (touch response and body bends), and antioxidant enzymes (superoxide dismutase [SOD] and catalase [CAT]) and malondialdehyde (MDA) levels in worms expressing TTR. First, we investigated the influence of santalol isomers on the reproduction and development of *C. elegans*. The results showed that expression of wild-type and V30M mutant TTR protein reduced progeny production and body length of *C. elegans*, while treatment with α-+β-santalol significantly reversed natural reproduction and development (*p* < 0.01) (Figures 7A,B). Next, we studied the effects of santalol isomers on chemotaxis and food sensing behavior, and they have also been reported to decline with age in *C. elegans* (Mohankumar et al., 2020). In FAP patients, the deposition of TTR aggregates around autonomic and peripheral neurons is linked with the loss of nociception, a process that detects noxious stimuli (Coelho et al., 2013). Similarly, the expression of TTR_V30M_ in body wall muscle cells induces nonautonomous sensory nociception defects and affects dendritic outgrowth in *C. elegans* (Madhivanan et al., 2018). TTR_WT_ and TTR_V30M_ worms pre-exposed to α-+β-santalol exhibit enhanced chemotaxis (Figure 7C) and food sensing behavior (Figure 7D) during the later adulthood stage (day 10; *p* < 0.01) compared to age-matched control worms, indicates the healthy status of sensory neurons. Consistent with the previous observation, however, the expression of TTR_WT_ and TTR_V30M_ significantly reduced memory and learning ability in *C. elegans* (*p* < 0.01) than non-TTR control and wild-type animals, suggesting the neurotoxic property of TTR aggregates (Figures 7C,D; Madhivanan et al., 2018). To test whether santalol isomers protect the other neurons from TTR-induced toxicity, we evaluated soft-touch sensing ability. In *C. elegans*, the ability to sense and respond to touch is largely controlled by mechanoreceptor neurons and their functions decrease with age (Pan et al., 2011) and TTR aggregation (Madhivanan et al., 2018). We observed that day 10 adulthood wild-type worms exhibited a reduction in age-dependent anterior and posterior touch response, while TTR expression further reduced the response to soft touch. However, α-+β-santalol treatment significantly improved the anterior and posterior touch response in worms expressing TTR_WT_ and TTR_V30M_ late in life (Figure 7E). To further investigate the effect of santalol isomers on motor activity, we compared the body bends (swimming vigor) between control and α-+β-santalol treated groups in old age (day 10). As shown in Figure 7F, the swimming vigor of worms maintaining the expression of TTR_WT_ and TTR_V30M_ in body wall muscle cells was significantly lower than that of their wild-type counterparts. Besides, santalol isomers treated TTR_WT_ and TTR_V30M_ worms displayed enhanced swimming vigor/body bends at old age than untreated controls. Interestingly, we found that the body bends of TTR expressing worms treated with α-+β-santalol are remarkably higher than wild-type worms exposed to similar concentrations of santalol isomers.

Previously, the relationship between TTR levels and reactive oxygen species (ROS) in the tissues of patients with TTR deposits and *C. elegans* was established (Ando et al., 1997; Tsuda et al., 2018). Santalol isomers also reportedly possess antioxidant activity by quenching the free radicals (Mohankumar et al., 2018). Therefore, we measured the ROS levels in TTR expressing worms in the presence and absence of α-+β-santalol using a ROS-specific fluorescent probe H_2_DCF-DA. Results showed that the TTR_WT_ and TTR_V30M_ worms showed higher intracellular ROS levels. In the presence of α-+β-santalol, however, the elevation in ROS levels observed in TTR_WT_ and TTR_V30M_ worms was significantly reduced to almost the control values (Figure 7G). We also examined the effect of santalol isomers on the activities of SOD, CAT, and MDA in TTR worms (Figure 7H). Day 5 adulthood worms expressing TTR_WT_ and TTR_V30M_ aggregates exhibited significantly reduced SOD and CAT activities compared to age-matched wild-type worms. Compared with the control, the SOD and CAT activities were restored and upregulated by α-+β-santalol treatment. Furthermore, the levels of lipid peroxidation, as evidenced by MDA levels, were 76.8 and 66.4% lower in α-+β-santalol-treated TTR_WT_ and TTR_V30M_ worms than those in untreated groups. Untreated worms from TTR_WT_ and TTR_V30M_ groups showed relatively higher levels of MDA compared to wild-type counterparts (*p* < 0.01), and the overproduction of MDA indicates oxidative damages (Figure 7H). These results clearly show that TTR aggregates generate intracellular ROS and that α-+β-santalol exhibited antioxidant activity in *C. elegans*. From these observations, it was apparent that the synergism between α- and β-santalol improved several healthspan measures in *C. elegans* models of TTR amyloidosis during their early and late in life.

## Conclusion

In summary, we have shown that santalol isomers are potent inhibitors of TTR amyloidogenesis. Santalol isomers effectively inhibit fibril formation and stabilize the native tetrameric form of TTR under denaturation conditions *in vitro*. We found that the combination of santalol isomers (α-+β-santalol) acts synergistically to prevent TTR-induced toxicity, lifespan reduction, and other functional deficits in *C. elegans*. Moreover, santalol isomers interact with TTR variants (TTR_WT_ and TTR_V30M_) and reduce the flexibility of the T_4_ binding cavity and the homotetramer interface, which in turn confers the conformational stability of TTR and prevents the dissociation of the tetramer and subsequent self-assembly into toxic aggregates. Santalol isomers inhibit TTR aggregation and help maintain proteostasis in *C. elegans* by activating the SKN-1/Nrf2 protective pathway, autophagy, and proteasome. SKN-1/Nrf2, autophagy, and proteasome are highly conserved between *C. elegans* and mammals, and hence it is not unfair to hypothesize that santalol isomers may also be protective against TTR-induced toxicity in patients with FAP, FAC, and/or SSA. Therefore, preclinical studies on mammalian models of TTR amyloidosis using a combination of santalol isomers are justified.

## Data Availability

The raw data supporting the conclusions of this article will be made available by the authors, without undue reservation.
