# Supplementary material for: Santalol Isomers Inhibit Transthyretin Amyloidogenesis and Associated Pathologies in Caenorhabditis elegans
Source: Front Pharmacol. 2022 Jun 16;13:924862. doi: 10.3389/fphar.2022.924862 (PMC9243336; doi:10.3389/fphar.2022.924862)
Supplement: Supplementary file 1 [file DataSheet1.pdf]

SUPPLEMENTARY MATERIAL

for

Santalol Isomers Inhibit Transthyretin Amyloidogenesis and Associated Pathologies in *Caenorhabditis elegans*

Amirthalingam Mohankumar [1, 2]*, Duraisamy Kalaiselvi [3], Govindhan Thiruppathi [2], Sivaramakrishnan Muthusaravanan [4], Subramaniam Vijayakumar [5], Rahul Suresh [6], Shinkichi Tawata [1]*, Palanisamy Sundararaj [2]*

[1]PAK Research Center, University of the Ryukyus, Okinawa, Japan

[2]Department of Zoology, Bharathiar University, Coimbatore, India

[3]Department of Agricultural Chemistry, Institute of Environmentally Friendly Agriculture, College of Agriculture and Life Science, Chonnam National University, Gwangju, South Korea

[4]Department of Biotechnology, Mepco Schlenk Engineering College, Sivakasi, India

[5]Department of Medical Physics, Bharathiar University, Coimbatore, India

[6]International Research Center of Spectroscopy and Quantum Chemistry—IRC SQC, Siberian Federal University, Krasnoyarsk, Russia

***Correspondence to,**

Amirthalingam Mohankumar (amkkmohan@gmail.com), Shinkichi Tawata (b986097@agr.u-ryukyu.ac.jp), Palanisamy Sundararaj (sunpalan@mail.com)

CONTENTS

1 Materials and methods **Page**

Isolation of santalol isomers ………………………………………………………... 3
C. elegans strain maintenance and age synchronization …………………………. 3
Food sensing behavior (basal slowing response to food) ………………………… 4
Chemotaxis assay ………………………………………………………………… 5
Measurement of body bends …………………………………………………...... 5
Determination of the antioxidant enzyme activities ……………………………… 5
Molecular modeling ……………………………………………………………… 6
TTR aggregation inhibition and stabilization by santalol isomers under *in vitro* conditions ………………………………………………………………………… 6

2 Figures

Figure 1. Santalol isomers and tolcapone over TTR_{WT} and TTR_{V30M} stability …... 8
Figure 2. Inhibitory effect of santalol isomers on the formation of TTR aggregates ……………………………………………………………………… 9
Figure 3. Lifespan analyses …………………………………………………… 10
Figure 4. The relative expression rate of individual subunits of 26S proteasome.... 11

3 Tables

Table 1. Inhibition of TTR fibril formation ……………………………………. 12
Table 2. Effect of santalol isomers on the aggregation of TTR variants in *C. elegans* ……………………………………………………………………………. 13
Table 3. Lifespan analyses ……………………………………………………... 14
Table 4. Synergistic effect of santalol isomers on the lifespan of *C. elegans* ……. 16
Table 5. Lifespan analyses ……………………………………………………... 17
References ……………………………………………………………………... 18

1. MATERIALS AND METHODS

1.1. Isolation of santalol isomers

The East Indian sandalwood oil was extracted from the heart-wood of plantation-grown *Santalum album* L. (Santalaceae) trees under Good Manufacturing Practice (GMP)/Good Laboratory Practice (GLP) regulatory guidelines was obtained from Quintis Forestry (Australia) Pty Ltd., (Perth, Western Australia). A second distillation under vacuum (rectification) was conducted to remove low-boiling santalenes that are typically present at low levels in sandalwood oil. The santalol isomers were purified from sandalwood oil by sequential column chromatography on silica gel (Daramwar et al., 2012), followed by supercritical fluid chromatography on chiral support (Averica Discovery Services, Worcester, MA, USA). The purity of isolated santalol isomers was determined by various spectral and analytical techniques, including gas chromatography-flame ionization detector (GC-FID), fourier-transform infrared spectroscopy (FTIR), nuclear magnetic resonance (^{1}H, ^{13}C, and 2D NMR), liquid chromatography-mass spectrometry (LC-MS), and elemental analysis. Certificate of analysis for sandalwood oil, α- and β-santalol can be found in the supporting information of our previous article (Mohankumar et al., 2018). Components of East Indian sandalwood oil were reported in our article (Mohankumar et al., 2019). Purified α- and β-santalol had 98.2% and 93.2% purity, respectively. The purified (93.2%) β-santalol also contains probably ~2.5% α-santalol, a small amount of (Z)-α-trans-bergamotol, epi β-santalol and solvents (mostly hexane), and ~0.23% water.

1.2. *C. elegans* strain maintenance and age synchronization

All the worm strains were maintained and propagated onto the nematode growth media (NGM) agar plates (17 g agar, 2.5 g casein peptone, 3 g NaCl, 1 mL of 1 M $CaCl_2$, 1 mL of 1 M $MgSO_4$, 1 mL of cholesterol [5 mg/mL], 1 mL nystatin [10 mg/ml], 25 mL potassium phosphate buffer, and distilled water) seeded with live *E. coli* OP50. For age

synchronization, gravid adult worms were collected from NGM plates, washed thrice, and the worms pellet was resuspended in 3.5 mL of M9 buffer (6g Na_2HPO_4, 3 g KH_2PO_4, 5 g NaCl, 0.25 g $MgSO_4 \cdot 7H_2O$, and distilled water). Afterward, freshly prepared 0.5 mL 5 N NaOH and 1 mL of household bleach (sodium hypochlorite/ NaOCl) were added to the worm suspension and vortexed for 6-10 min until the bodies of the entire worms dissolved. The eggs were then washed 4-5 times with M9 buffer to completely remove traces of NaOH and bleach. After the final wash, eggs were resuspended in M9 buffer and incubated at 20°C to favor the hatching (Brenner, 1974; Stiernagle, 2006).

TTR expressing *C. elegans* strains *viz.,* KXK1069 (zyeEx1069[pCKX3307(*unc-54p::nsTTR*$_{WT}$*::EGFP*)]), KXK1070 (zyeEx1070[pCKX3311(*unc-54p::nsTTR*$_{1\text{-}80}$*::EGFP*)]), KXK1073 (zyeEx1073[pCKX3303(*unc-54p::nsTTR*$_{81\text{-}127}$*::EGFP*)]), KXK1076 (zyeEX1076[pCKX3316(*unc-54p::nsEGFP*)]), KXK1078 (zyeEx1078[pCKX3309(*unc-54p::nsTTR*$_{V30M}$*::EGFP*)]), and KXK1082 (zyeEx1082[pCKX3313(*unc-54p::nsTTR*$_{49\text{-}127}$*::EGFP*)]) were maintained by picking the GFP positive animals (Tsuda et al., 2018).

1.3. Food sensing behavior (basal slowing response to food)

Assay plates were prepared in a 9 cm diameter Petri dish by spreading *E. coli* OP50 overnight in a ring with an inner diameter of ~1 cm and an outer diameter of ~8 cm on NGM agar. After treatment with santalol isomers, the nematodes (n=30–40 per treatment) were washed with M9 buffer and released to the center of the NGM agar plate spotted with or without *E. coli* OP50 lawn. After five minutes, the body bends of each nematode were measured for 1 min in the presence or absence of food, and the slowing response was calculated using the following formula;

$$\text{Slowing rate} = (N_{\text{without food}} - N_{\text{with food}}) / N_{\text{without food}}$$

Where N represents the total number of body bends in the presence or absence of a bacterial food source.

1.4. Chemotaxis assay

Chemotaxis assay was performed according to Bargmann et al., method (Bargmann et al., 1993). Briefly, the age synchronized worms (n=100-120 worms per treatment) were treated with α- and β-santalol. Day 5 and day 10 adulthood stage worms were transferred to chemotaxis plates divided into four equal quadrants (A1, B1, A2, B2) carrying 10 μL attractant (1 M sodium acetate) on one side (A1, A2) and 10 mL distilled water on the other side (B1, B2). 25 mM sodium azide was spotted on each side to paralyze the attracted worms towards the region. The worms were released at the center of the plates and incubated at 20°C for 90 min, and the chemotaxis index (CI) was calculated using the following formula;

Chemotaxis index (CI) = (A1 + A2) - (B1 + B2) / *N*

Where A1 and A2 represent worms in the attractant region, B1 and B2 represent worms in the control region, and *N* represents the total number of worms. Three independent trials were performed with appropriate replicates.

1.5. Measurement of body bends

Control and treated worms were washed twice with M9 buffer and released onto the unseeded NGM plates to crawl for 5 min. Individual worms were then transferred to 24-well microtiter plates containing 1 mL of M9 buffer. After 1 min recovery period, the number of body bends was scored for 30 seconds using a stereo zoom microscope. The reciprocating motion of bending at the mid-body of *C. elegans* was considered as body bend.

1.6. Determination of the antioxidant enzyme activities

Worms were treated as said in the lifespan assay. After treatment, control and treated worms (500 individuals per experiment) were homogenized, and the activities of the antioxidant enzymes superoxide dismutase (SOD), catalase (CAT), and malondialdehyde (MDA) were measured according to the instructions provided by the manufacturer. Total

protein content was determined by the BCA assay kit, and the results were normalized by total protein contents represented as U/mg protein or nmol/mg protein.

1.7. Molecular modeling

Molecular dynamics simulation was performed with GROMACS 2016.4 (Hess et al., 2008) with a CHARMM36 force field and TIP3P water model (Jorgensen et al., 1983; Vanommeslaeghe et al., 2009). Periodic boundary conditions were applied in three dimensions, and long-range electrostatic interactions were treated by the particle mesh Ewald's method (Essmann et al., 1995). The pressure was controlled at 1 atm using Parrinello-Rehman barostat, and the temperature was maintained at 310 K with Langevin's dynamics (Martyna et al., 1994). The short-range and long-range interactions were truncated at 1.2 Å and 1.4 Å, and the LINCS algorithm was used to constrain the bond involving hydrogen atoms (Hess et al., 1997). Equilibration was carried out in NVT, followed by an NPT ensemble with a time step of one fs. The production run with a time step of 2 fs for 50 ns was performed in the NPT ensemble, and snapshots were collected every 100 ps intervals. Molecular visualization and analyses were performed with the Visual Molecular Dynamics (VMD) and UCSF Chimera package (Humphrey et al., 1996; Pettersen et al., 2004).

1.8. TTR aggregation inhibition and stabilization by santalol isomers under *in vitro* conditions

To measure the *in vitro* TTR aggregation inhibition, 7 µL of TTR solution containing TTR_{WT} or $TTRV_{30M}$ in 10 mM sodium phosphate, 1 mM EDTA, and 100 mM KCl (pH 7.6) were incubated with different concentrations of santalol isomers at 37°C for 1 h. After the incubation, the pH of the solution was reduced to 4.2 with 100 mM KCl and 400 mM sodium acetate. This mixture was then incubated at 37°C for 72 h, and the inhibition of fibril formation was measured with a microplate reader at 340 nm (Lai et al., 1996; Dolado et al., 2005; Sant'Anna et al., 2016). To examine the TTR stabilization by santalol isomers, TTR (1

μg in PBS) was incubated with and without α- and β-santalol for 1 h. Denaturation was induced by adding 8 M urea into the protein mixture and subsequently incubated for 72 h at room temperature. The Trp fluorescence was measured at 280 nm/355 nm (excitation/emission) in a Spectrofluorometer as described (Sant'Anna et al., 2016).

2. FIGURES

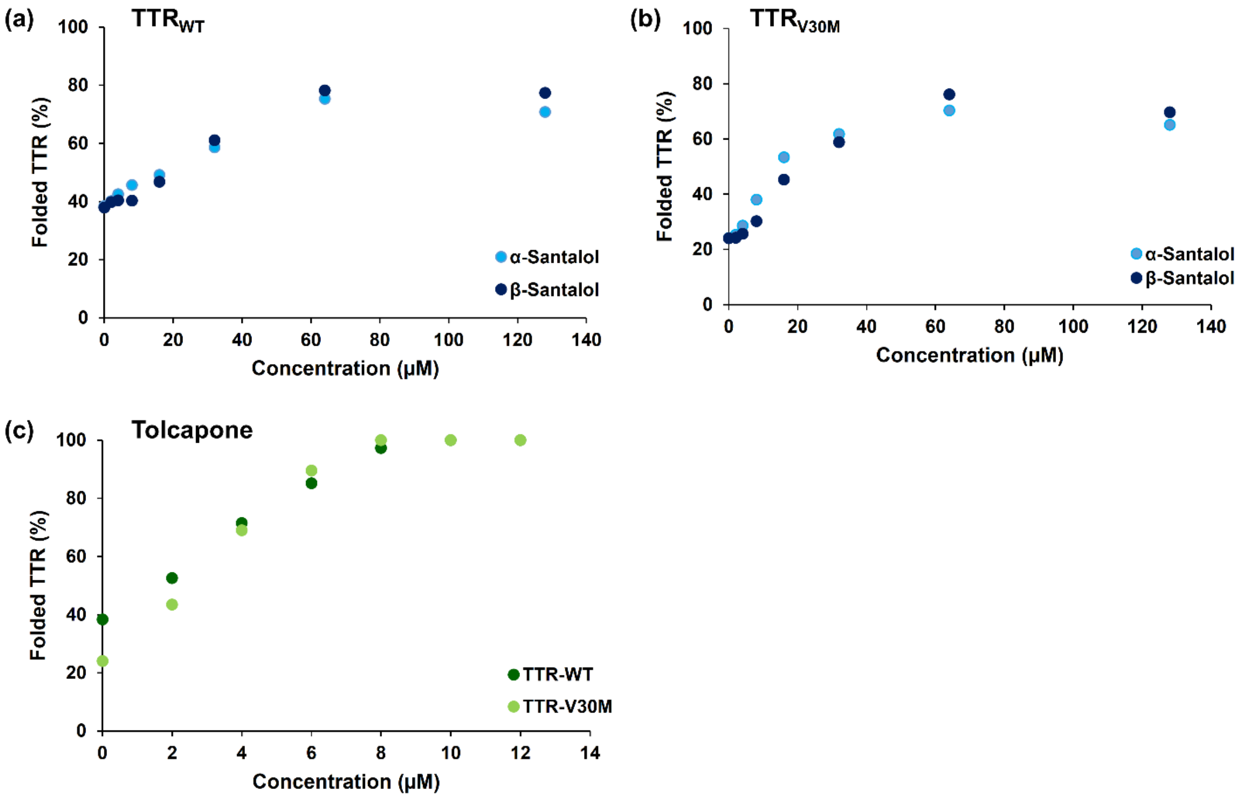

Figure S1. Effect of santalol isomers and tolcapone over TTR_{WT} and TTR_{V30M} stability. (**a**) TTR_{WT} and (**b**) TTRV30M was incubated with several concentrations of santalol isomers and (**c**) tolcapone, and denaturation was induced by the addition of 8 M urea. Stabilizing effect of santalol isomers and tolcapone was analyzed by measuring Trp fluorescence intensity at 280 nm/355 nm (excitation/emission). Combined data from four independent biological trials were presented, each performed in triplicate.

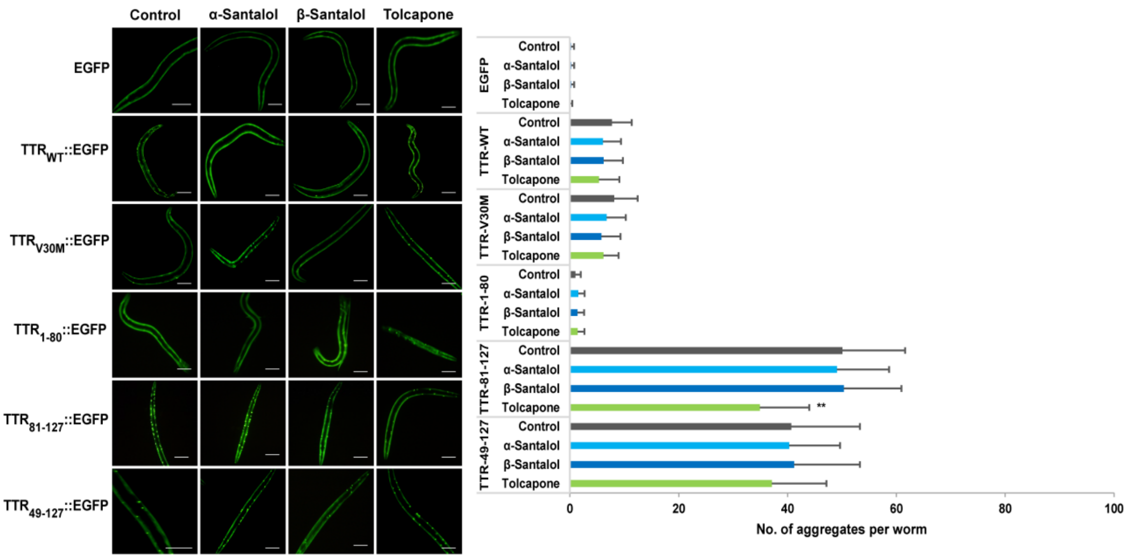

Figure S2. Inhibitory effect of santalol isomers on the formation of TTR aggregates in day 2 adulthood worms. *C. elegans* strains expressing EGFP, TTR_{WT}::EGFP, TTR_{V30M}::EGFP, TTR_{1-80}::EGFP, TTR_{81-127}::EGFP, and TTR_{49-127}:EGFP were used. The scale bar represents 100 µm. Data are presented as mean ± SD; **$p<0.01$ vs. control. See supporting information **Table S1** for more detailed statistics.

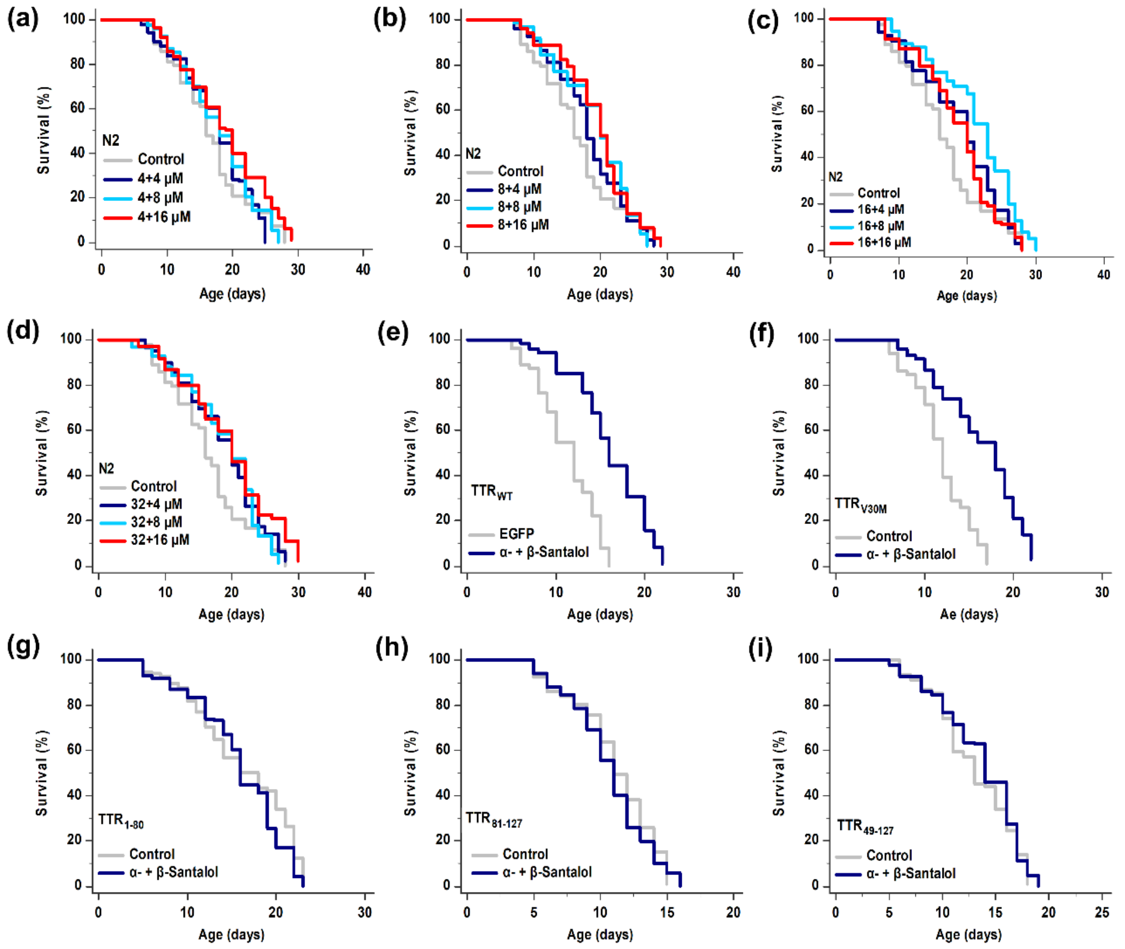

Figure S3. Lifespan analyses. (a-d) Synergistic effect of santalol isomers on the lifespan of wild-type worms at standard laboratory conditions. Effect of α-+β-santalol on the lifespan of worms expressing (e) TTR$_{WT}$, (f) TTR$_{V30M}$, (g) TTR$_{1-80}$, (h) TTR$_{81-127}$, and (i) TTR$_{49-127}$ fragments in body wall muscle cells. Combined data of three independent biological trials were presented. See supporting information **Table S3** and **Table S4** for statistical details of lifespan analyses.

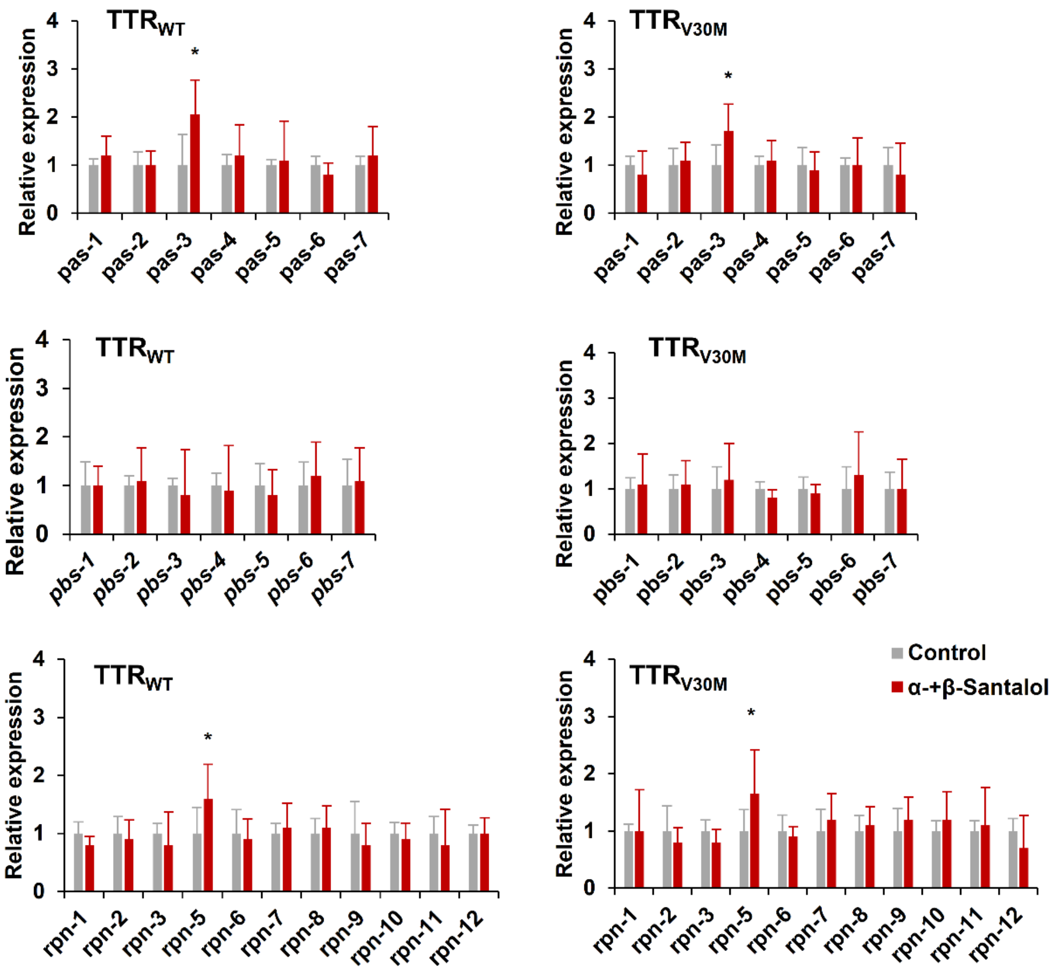

Figure S4. The relative expression rate of individual subunits of 26S proteasome in TTR_{WT} (left) and TTR_{V30M} (right) worms treated with α-+β-santalol. *$p<0.05$ vs control.

3. TABLES

Table S1. Inhibition of TTR fibril formation under acidic denaturation conditions (pH 4.2, 72 h) in the presence of santalol isomers or tolcapone

**Treatment (μM)**		**TTR_{WT}**		**TTR_{V30M}**	
		**% Fibril formation**	**EC_{50} (μM)**	**% Fibril formation**	**EC_{50} (μM)**
α-Santalol	2	81.3	36.7±1.2	91.7	44.3±3.5
	4	69.7		80.3	
	8	61.3		70.7	
	16	41.6		52.0	
	32	31.4		32.4	
	64	25.3		23.0	
	128	11.2		13.0	
β-Santalol	2	92.3	31.1±2.4	93.7	34.5±2.8
	4	78.6		82.0	
	8	58.3		64.0	
	16	38.0		31.7	
	32	12.0		20.3	
	64	8.6		14.0	
	128	8.3		8.6	
Tolcapone	2	70.6	4.6±0.5	76.7	4.8±0.7
	4	49.5		53.3	
	6	31.7		27.0	
	8	10.4		9.4	
	10	0		1.3	
	12	0		0	
	14	0		0	

% Fibril formation represents the extent of TTR_{WT} or TTR_{V30M} in the presence of santalol isomers or tolcapone compared to untreated TTR_{WT} and TTR_{V30M} (100% fibril formation).

Table S2. Effect of santalol isomers on the aggregation of TTR variants in *C. elegans* across various developmental stages

Genotype	Treatment	No. of TTR aggregates per worm (n=40)			
		Day 2		Day 6	
		No. of aggregates ± SD	% Change	No. of aggregates ± SD	% Change
EGFP	Control	0.23±0.50		0.20±0.48	
	α-Santalol	0.27±0.52 ns	(+) 14.29	0.37±0.67 ns	(+) 83.33
	β-Santalol	0.27±0.52 ns	(+) 14.29	0.17±0.38 ns	(-) 16.67
	Tolcapone	0.07±0.37 ns	(-) 71.43	0.13±0.35 ns	(-) 33.33
TTR_{WT}::EGFP	Control	7.73±3.62		32.03±10.44	
	α-Santalol	6.07±3.32	(-) 21.55	21.37±6.53**	(-) 33.30
	β-Santalol	6.20±3.54 ns	(-) 19.83	19.17±7.65**	(-) 40.17
	Tolcapone	5.33±3.78 ns	(-) 31.03	17.23±4.07**	(-) 46.20
TTR_{V30M}::EGFP	Control	8.13±4.30 ns		26.23±4.88	
	α-Santalol	6.77±3.50ns	(-) 16.80	17.10±4.74**	(-) 34.82
	β-Santalol	5.77±3.53ns	(-) 29.10	15.70±3.44**	(-) 40.15
	Tolcapone	6.17±2.76ns	(-) 24.18	15.83±5.56**	(-) 39.64
TTR_{1-80}::EGFP	Control	1.00±2.60		2.60±1.65	
	α-Santalol	1.53±1.17ns	(+) 53.33	2.17±1.46 ns	(-) 16.67
	β-Santalol	1.40±±1.22 ns	(+) 40.00	2.37±1.63 ns	(-) 8.97
	Tolcapone	1.43±1.25 ns	(+) 43.33	3.10±1.69 ns	(-) 19.23
TTR_{81-127}::EGFP	Control	50.10±11.51		76.90±19.62	
	α-Santalol	49.10±9.54 ns	(-) 2.00	75.33±14.80 ns	(-) 2.04
	β-Santalol	50.33±10.61 ns	(+) 0.47	75.73±18.57 ns	(-) 1.52
	Tolcapone	34.90±9.08**	(-) 30.34	39.97±.74**	(-) 48.03
TTR_{49-127}::EGFP	Control	40.67±12.66		88.97±19.16	
	α-Santalol	40.30±9.36 ns	(-) 0.90	87.30±11.91 ns	(-) 1.87
	β-Santalol	41.20±12.12 ns	(+) 1.31	8753±12.28 ns	(-) 1.61
	Tolcapone	37.13±10.00 ns	(-) 8.69	84.07±14.66 ns	(-) 5.51

Data are presented as mean ± SD; ns-not significant, *p<0.05, and **p<0.01 vs. control.

Table S3. Lifespan analyses

Genotype	Treatment	Mean survival (Mean±SEM)	Maximum lifespan (days)	Sample size (N)	Censored	% Change	*p* value
Wild-N2	Control	16.133±0.480	25	128	16		
EGFP	Control	15.937±0.440	24	122	11	(-) 1.21	0.3942
TTR_{WT}	EGFP	15.937±0.440	24	122	11		
	Control	11.273±0.278	16	136	7	(-) 29.27	0.0001#
	α-Santalol	14.424±0.402	20	113	3	(+) 27.95	0.0001*
	β-Santalol	14.982±0.454	22	119	8	(+) 32.90	0.0001*
	Tolcapone	14.790±0.473	22	124	6	(+) 31.20	0.0001*
TTR_{V30M}	EGFP	15.937±0.440	24	122	11		
	Control	11.909±0.269	17	137	5	(-) 25.27	0.0001#
	α-Santalol	14.389±0.339	21	151	10	(+) 20.82	0.0001*
	β-Santalol	15.215±0.344	20	145	16	(+) 27.76	0.0001*
	Tolcapone	14.650±0.458	21	114	7	(+) 23.02	0.0001*
TTR_{1-80}	EGFP	15.937±0.440	24	122	11		
	Control	16.255±0.451	23	153	9	(+) 2.00	0.2259#
	α-Santalol	15.750±0.414	22	138	7	(-) 3.11	0.0958
	β-Santalol	15.950±0.414	23	162	11	(-) 1.88	0.1264
	Tolcapone	16.788±0.480	24	119	7	(+) 3.28	0.4205
TTR_{81-127}	EGFP	15.937±0.440	24	122	11		
	Control	11.108±0.254	15	144	4	(-) 30.30	0.0001#
	α-Santalol	11.205±0.254	16	153	9	(+) 0.87	0.5076
	β-Santalol	10.888±0.258	16	134	3	(-) 1.98	0.5074
	Tolcapone	15.736±0.382	22	140	6	(+) 41.66	0.0001*
TTR_{49-127}	EGFP	15.937±0.440	24	122	11		
	Control	13.097±0.316	18	136	9	(-) 17.82	0.0001#
	α-Santalol	13.011±0.278	18	144	11	(-) 0.68	0.3274
	β-Santalol	13.304±0.315	18	130	7	(+) 1.58	0.6365
	Tolcapone	13.624±0.392	19	104	9	(+) 4.02	0.0906
TTR_{WT}	Control	11.273±0.278	16	136	7		
	α-+β-Santalol	16.124±0.378	22	121	7	(+) 43.03	0.0001*
TTR_{V30M}	Control	11.909±0.269	17	137	5		
	α-+β-Santalol	16.355±0.383	22	142	14	(+) 37.33	0.0001*
TTR_{1-80}	Control	16.255±0.451	23	153	9		
	α-+β-Santalol	15.860±0.447	23	132	15	(-) 2.43	0.0826
TTR_{81-127}	Control	11.108±0.254	15	144	4		
	α-+β-Santalol	10.716±0.243	16	149	11	(-) 3.53	0.2477
TTR_{49-127}	Control	13.097±0.316	18	136	9		
	α-+β-Santalol	13.635±0.323	19	137	8	(+) 4.11	0.2497
Wild-N2	Control	18.096±0.447	28	157	9		
	Tolcapone 2 µM	18.518±0.425	28	172	12	(+) 2.33	0.5247
	Tolcapone 4 µM	19.086±0.522	30	146	9	(+) 5.47	0.0310
	Tolcapone 6 µM	21.937±0.456	30	176	7	(+) 21.23	0.0001*
	Tolcapone 8 µM	20.594±0.344	25	162	7	(+) 13.80	0.0046
	Tolcapone 10 µM	19.506±0.402	25	141	8	(+) 7.79	0.2592

Genotype	Treatment	Mean survival (Mean±SEM)	Maximum lifespan (days)	Sample size (N)	Censored	% Change	*p* value
TTR_{WT}	Tolcapone 6 μM	14.790±0.473	22	124	6	(+) 31.20	0.0001*
TTR_{V30M}	Tolcapone 6 μM	14.650±0.458	21	114	7	(+) 23.02	0.0001*
TTR_{1-80}	Tolcapone 6 μM	16.788±0.480	24	119	7	(+) 3.28	0.4205
TTR_{81-127}	Tolcapone 6 μM	15.736±0.382	22	140	10	(+) 41.66	0.0001*
TTR_{49-127}	Tolcapone 6 μM	13.624±0.392	19	104	9	(+) 4.02	0.0906

Combined data of three independent biological trials were presented. Data were analyzed using the Kaplan−Meier survival method, and the significance level was estimated by the log-rank (Mantel−Cox) test. *p<0.0001 vs. control group; #p<0.0001 vs. EGFP.

Table S4. Synergistic effect of santalol isomers on the lifespan of wild-type *C. elegans*

Treatment	Mean survival (Mean±SEM)	Maximum lifespan (days)	Sample size (N)	Censored	% Change	*p* value
Control	16.653±0.534	28	128	16		
4 µM α-San.+4 µM β-San.	17.463±0.466	25	142	9	(+) 4.86	0.5534
4 µM α-San.+8 µM β-San.	17.872±0.507	27	123	8	(+) 7.32	0.2606
4 µM α-San.+16 µM β-San.	18.952±0.546	29	139	9	(+) 13.81	0.0014
8 µM α-San.+4 µM β-San.	18.232±0.467	28	134	7	(+) 9.48	0.1048
8 µM α-San.+8 µM β-San.	19.236±0.438	27	156	12	(+) 15.51	0.0034
8 µM α-San.+16 µM β-San.	19.561±0.463	29	131	4	(+) 17.46	0.0002
16 µM α-San.+4 µM β-San.	18.893±0.546	28	125	8	(+) 13.45	0.0061
16 µM α-San.+8 µM β-San.	21.311±0.529	30	131	5	(+) 27.97	0.0001*
16 µM α-San.+16 µM β-San.	18.704±0.473	28	13	10	(+) 12.32	0.0124
32 µM α-San.+4 µM β-San.	18.950±0.488	28	147	11	(+) 13.79	0.0025
32 µM α-San.+8 µM β-San.	18.949±0.470	27	153	16	(+) 13.79	0.0070
32 µM α-San.+16 µM β-San.	19.820±0.582	30	135	12	(+) 19.02	0.0001*

Combined data of three independent biological trials were presented. Data were analyzed using the Kaplan−Meier survival method, and the significance level was estimated by the log-rank (Mantel−Cox) test. *p<0.0001 vs. control group. α-San., α-Santalol; β-San., β-Santalol.

Table S5. Lifespan analyses

Genotype	Treatment	Mean survival (Mean±SEM)	Maximum lifespan (days)	Sample size (N)	Censored	% Change	*p* value
TTR_{WT}	Control	11.273±0.278	16	136	7		
	α-+β-Santalol	16.124±0.378	22	121	7	(+) 43.03	0.0001*
	skn-1 RNAi/ α-+β-Santalol	11.606±0.298	18	126	5	(-) 28.02	0.0001#
	lgg-1 RNAi/ α-+β-Santalol	12.014±0.316	19	144	5	(-) 25.49	0.0001#
	Vector control	11.225±0.279	16	133	10	(-) 0.43	0.9429*
TTR_{V30M}	Control	11.909±0.269	17	137	5		
	α-+β-Santalol	16.355±0.383	22	142	14	(+) 37.33	0.0001*
	skn-1 RNAi/ α-+β-Santalol	12.905±0.300	18	152	9	(-) 21.09	0.0001#
	lgg-1 RNAi/ α-+β-Santalol	12.248±0.328	19	130	3	(-) 25.11	0.0001#
	Vector control	11.665±0.297	17	130	9	(-) 2.05	0.8452*
bec-1	Control	14.962±0.329	22	123	5		
	α-+β-Santalol	15.207±0.331	23	140	11	(+) 1.64	0.3404*
hlh-30	Control	15.509±0.310	21	142	7		
	α-+β-Santalol	17.278±0.340	24	154	13	(+) 11.41	0.0001*

Combined data of three independent biological trials were presented. Data were analyzed using the Kaplan−Meier survival method, and the significance level was estimated by the log-rank (Mantel−Cox) test. *p<0.0001 vs. control group, #p<0.0001 vs. α-+β-santalol treated groups.

REFERENCES

Bargmann, C. I., Hartwieg, E., and Horvitz, H. R. (1993). Odorant-Selective Genes and Neurons Mediate Olfaction in *C. elegans*. *Cell* 74, 515–527. doi:10.1016/0092-8674(93)80053-H.

Brenner, S. (1974). The Genetics of *Caenorhabditis elegans*. *Genetics* 77, 71–94. doi:10.1002/cbic.200300625.

Daramwar, P. P., Srivastava, P. L., Priyadarshini, B., and Thulasiram, H. V. (2012). Preparative Separation of α- and β-Santalenes and (Z)-α- and (Z)-β-Santalols Using Silver Nitrate-Impregnated Silica Gel Medium Pressure Liquid Chromatography and Analysis of Sandalwood Oil. *Analyst* 137, 4564. doi:10.1039/c2an35575b.

Dolado, I., Nieto, J., Saraiva, M. J. M., Arsequell, G., Valencia, G., and Planas, A. (2005). Kinetic Assay for High-Throughput Screening of *in vitro* Transthyretin Amyloid Fibrillogenesis Inhibitors. *J. Comb. Chem.* 7, 246–252. doi:10.1021/cc049849s.

Essmann, U., Perera, L., Berkowitz, M. L., Darden, T., Lee, H., and Pedersen, L. G. (1995). A Smooth Particle Mesh Ewald Method. *J. Chem. Phys.* 103, 8577–8593. doi:10.1063/1.470117.

Hess, B., Bekker, H., Berendsen, H. J. C., and Fraaije, J. G. E. M. (1997). LINCS: A Linear Constraint Solver for Molecular Simulations. *J. Comput. Chem.* 18, 1463–1472. doi:10.1002/(SICI)1096-987X(199709)18:12<1463::AID-JCC4>3.0.CO;2-H.

Hess, B., Kutzner, C., van der Spoel, D., and Lindahl, E. (2008). GROMACS 4: Algorithms for Highly Efficient, Load-Balanced, and Scalable Molecular Simulation. *J. Chem. Theory Comput.* 4, 435–447. doi:10.1021/ct700301q.

Humphrey, W., Dalke, A., and Schulten, K. (1996). VMD: Visual Molecular Dynamics. *J. Mol. Graph.* 14, 33–38. doi:10.1016/0263-7855(96)00018-5.

Jorgensen, W. L., Chandrasekhar, J., Madura, J. D., Impey, R. W., and Klein, M. L. (1983). Comparison of Simple Potential Functions for Simulating Liquid Water. *J. Chem. Phys.* 79, 926–935. doi:10.1063/1.445869.

Lai, Z., Colón, W., and Kelly, J. W. (1996). The Acid-Mediated Denaturation Pathway of Transthyretin Yields a Conformational Intermediate That Can Self-Assemble into Amyloid. *Biochemistry* 35, 6470–6482. doi:10.1021/bi952501g.

Martyna, G. J., Tobias, D. J., and Klein, M. L. (1994). Constant Pressure Molecular Dynamics Algorithms. *J. Chem. Phys.* 101, 4177–4189. doi:10.1063/1.467468.

Mohankumar, A., Kalaiselvi, D., Levenson, C., Shanmugam, G., Thiruppathi, G., Nivitha, S., et al. (2019). Antioxidant and Stress Modulatory Efficacy of Essential Oil Extracted

from Plantation-Grown *Santalum album* L. *Ind. Crops Prod.* 140, 111623. doi:10.1016/j.indcrop.2019.111623.

Mohankumar, A., Shanmugam, G., Kalaiselvi, D., Levenson, C., Nivitha, S., Thiruppathi, G., et al. (2018). East Indian Sandalwood (*Santalum album* L.) Oil Confers Neuroprotection and Geroprotection in *Caenorhabditis elegans* via Activating SKN-1/Nrf2 Signaling Pathway. *RSC Adv.* 8, 33753–33774. doi:10.1039/C8RA05195J.

Pettersen, E. F., Goddard, T. D., Huang, C. C., Couch, G. S., Greenblatt, D. M., Meng, E. C., et al. (2004). UCSF Chimera: A Visualization System for Exploratory Research and Analysis. *J. Comput. Chem.* 25, 1605–1612. doi:10.1002/jcc.20084.

Sant'Anna, R., Gallego, P., Robinson, L. Z., Pereira-Henriques, A., Ferreira, N., Pinheiro, F., et al. (2016). Repositioning Tolcapone as a Potent inhibitor of Transthyretin Amyloidogenesis and Associated Cellular Toxicity. *Nat. Commun.* 7, 10787. doi:10.1038/ncomms10787.

Stiernagle, T. (2006). Maintenance of *C. elegans*. *WormBook*. doi:10.1895/wormbook.1.101.1.

Tsuda, Y., Yamanaka, K., Toyoshima, R., Ueda, M., Masuda, T., Misumi, Y., et al. (2018). Development of Transgenic *Caenorhabditis elegans* Expressing Human Transthyretin as a Model for Drug Screening. *Sci. Rep.* 8, 17884. doi:10.1038/s41598-018-36357-5.

Vanommeslaeghe, K., Hatcher, E., Acharya, C., Kundu, S., Zhong, S., Shim, J., et al. (2009). CHARMM General Force Field: A Force Field for Drug-Like Molecules Compatible with the CHARMM All-Atom Additive Biological Force Fields. *J. Comput. Chem.*, 31(4), 671-690. doi:10.1002/jcc.21367.
